# Supplementary material for: Complementary Ribo-seq approaches map the translatome and provide a small protein census in the foodborne pathogen Campylobacter jejuni
Source: Nat Commun. 2025 Mar 30;16:3078. doi: 10.1038/s41467-025-58329-w (PMC11955535; doi:10.1038/s41467-025-58329-w)
Supplement: Supplementary file 1 — Supplementary Information [file 41467_2025_58329_MOESM1_ESM.pdf]

Supplementary Information

Complementary Ribo-seq approaches map the translatome and provide a small protein census in the foodborne pathogen *Campylobacter jejuni*

Kathrin Froschauer*, Sarah L. Svensson*, Rick Gelhausen, Elisabetta Fiore, Philipp Kible, Alicia Klaude, Martin Kucklick, Stephan Fuchs, Florian Eggenhofer, Chao Yang, Daniel Falush, Susanne Engelmann, Rolf Backofen, & Cynthia M. Sharma

This document contains:

Supplementary Figures 1-15

Supplementary Table 1

Online Methods

Supplementary References

*equally contributing authors

Supplementary Figures

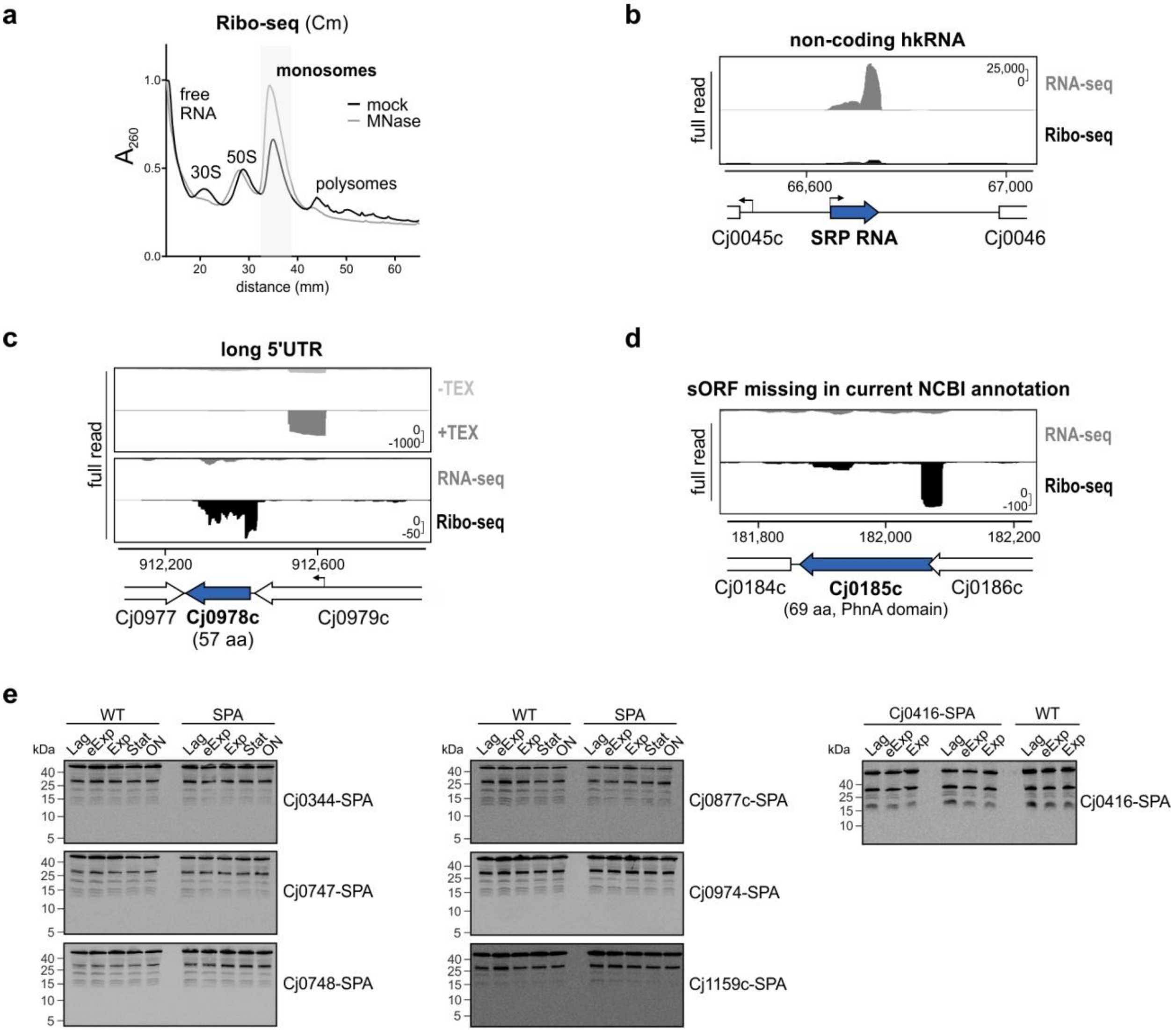

Supplementary Figure 1. A *C. jejuni* Ribo-seq protocol captures translating ribosomes and distinguishes coding from non-coding features. a, Polysome profiles for mock- and micrococcal nuclease (MNase)-treated lysates from *C. jejuni* strain NCTC11168 WT. Cells were harvested from log phase cultures in rich medium. Lysates were separated on 10-55% sucrose density gradients by ultracentrifugation and the RNA content of fractions was measured (A_{260}) to identify complexes. Shaded region: fractions harvested for library preparation. Representative of *n* = 3 independent experiments. **b,** cDNA reads for the non-coding housekeeping RNA (hkRNA) component of the signal recognition particle (SRP) are mainly restricted to RNA-seq, rather than Ribo-seq, libraries. **c,** MNase digestion of unprotected RNA validates a long (~200 nt[1]) 5′UTR of sORF Cj0978c (57 aa). **d,** Ribo-seq suggests that Cj0185c, absent from the current *C. jejuni* NCTC11168 NCBI annotation, is translated from an operon with Cj0184c and Cj0186c. For all screenshots, y-axis scales represent rpm (reads per million) and coverage is representative of *n* = 3 independent experiments. **e,** Western blots for C-

terminal SPA epitope-tagged annotated sORFs that could not be detected on western blots in rich medium. Visible bands represent unspecific binding of the anti-FLAG antibody. eExp: early exponential. Stat: stationary. ON: overnight. Untagged WT: antibody (anti-FLAG) control. Representative Western blot of $n = 2$ independent experiments. Related to main **Fig. 1e**. Source data are provided as a Source Data file.

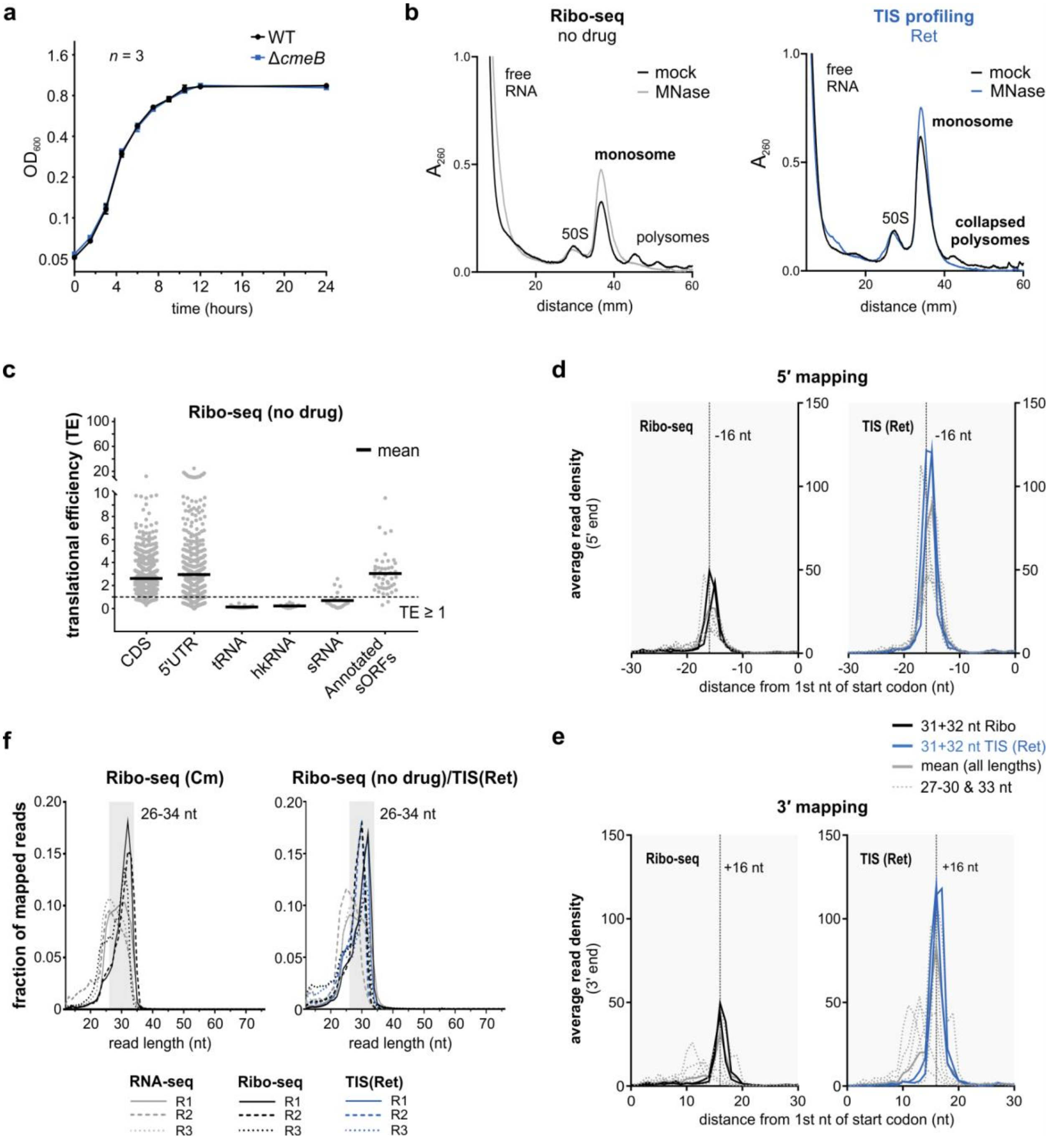

Supplementary Figure 2. Establishing Ret-mediated TIS profiling in *C. jejuni*. a, Growth of a *C. jejuni* Δ*cmeB* mutant vs. the parental WT strain under standard conditions in rich media. Ribo-seq cultures were harvested at OD_{600} ~0.4. Error bars indicate the standard deviation of *n* = 3 independent cultures. **b,** Polysome profiles (-/+ MNase treatment) for untreated (no drug; *left*) or Ret-treated (12.5 µg/ml, 10 min; *right*) *C. jejuni* Δ*cmeB*. Lysates were separated on a 10-55% sucrose density gradient by ultracentrifugation and the RNA content of fractions was measured (A_{260}) to identify complexes. Representative of *n* = 3 independent experiments. **c,** Translational efficiency (TE: Ribo-seq/RNA-seq) for feature classes in the *C. jejuni* annotation[1,2] of the no-drug control (no Cm) of

the TIS(Ret) experiment. A total RNA RPKM of ≥ 30 was required. CDS: coding sequence. hk: housekeeping. **d, e** Metagene analysis of ribosome occupancy (no drug vs. Ret) at start codons for indicated read lengths (5′ read end mapping, panel **d**; 3′, panel **e**). Related to main **Fig. 2a**. Solid black: 31 or 32 nt-long reads for Ribo-seq (no drug). Solid blue: 31 or 32 nt reads for TIS profiling (Ret). Solid grey: mean of all lengths. Dashed grey lines: all other lengths (27-30 and 33 nt). **f,** Read length distribution for libraries from Ribo-seq (Cm), Ribo-seq (no drug), and TIS(Ret) experiments. The size-selected lengths (26-34 nt) are highlighted with grey boxes. Grey lines: RNA-seq; black lines: Ribo-seq; blue lines: TIS(Ret). Solid line: replicate 1; dashed line: replicate 2; dotted line: replicate 3. Source data are provided as a Source Data file.

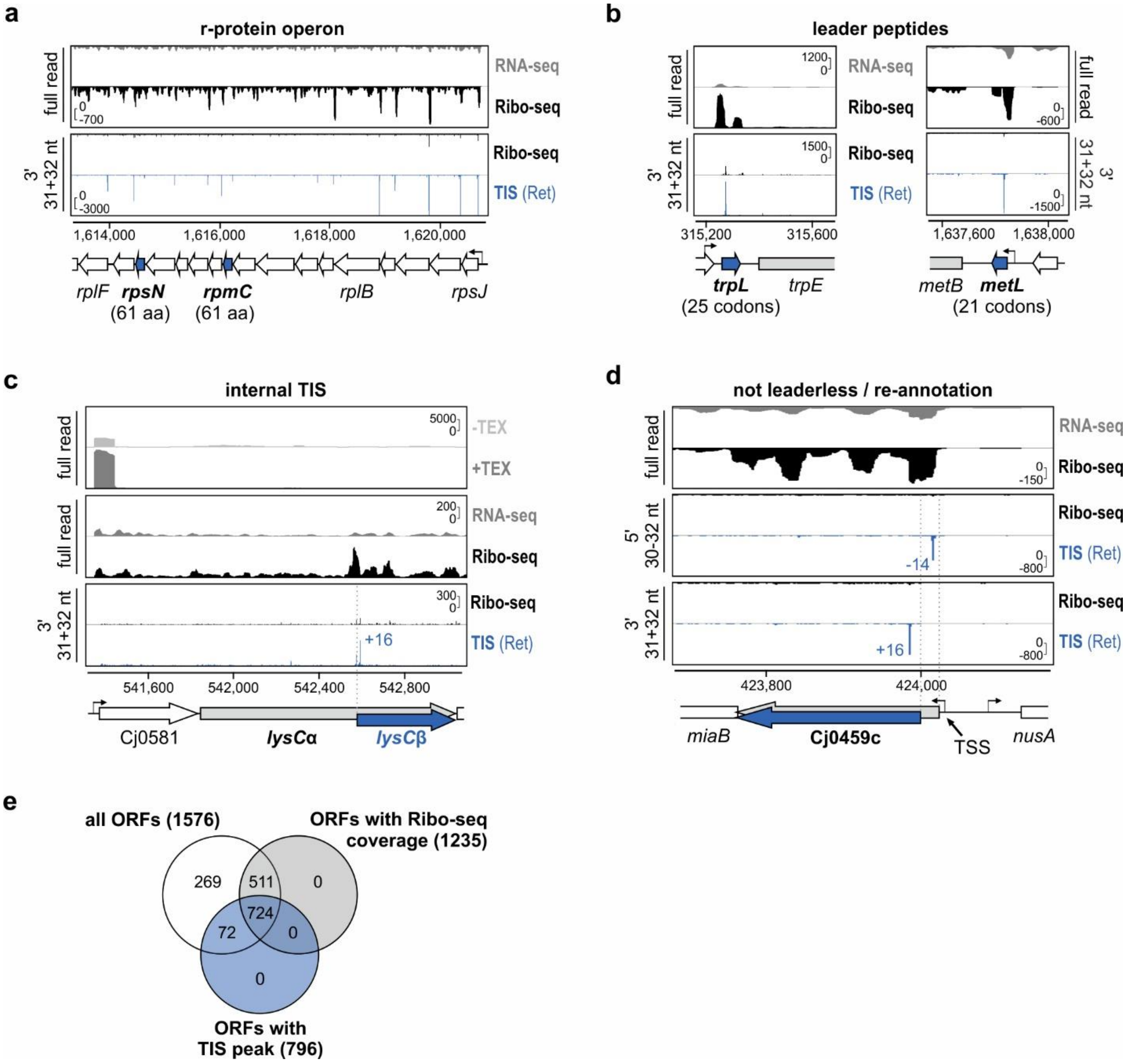

Supplementary Figure 3. Ret-mediated TIS profiling reveals *C. jejuni* start codons. a, Ribosome occupancy across a ribosomal protein-encoding (r-protein) operon. Two small sORFs (*rpsN* & *rpmC*, both 61 aa) are highlighted. Top two tracks: full read coverage for paired RNA-seq and conventional Ribo-seq libraries. Bottom two tracks: 3'-end coverage for 31 + 32 nt reads for Ribo-seq and TIS libraries. Enriched peaks in TIS (Ret) vs. Ribo-seq (arrows) suggest translation initiation sites. **b,** TIS detection of two previously predicted *C. jejuni* leader peptides/uORFs in amino acid biosynthesis operons (TrpL, *left;* MetL, *right*)[3]. Related to main **Fig. 2b**. **c,** TIS data reveals a potential 156 aa LysCβ isoform encoded in *lysC* (LysCα, Cj0582, 401 aa, grey) generated via internal translation initiation as reported in other bacteria[4]. +16 nt: TIS peak. The *lysCβ* ORF (blue) was also predicted from Ribo-seq data. **d,** TIS profiling suggests the predicted[3] leaderless gene Cj0459c (in grey, 5′UTR: 6 nt) should be re-annotated (indicated as blue arrow) as a shorter ORF leadered with a 30 nt 5′UTR. Both 5′ and 3′ end coverage was used. For all screenshots, bent arrows: TSS based on dRNA-seq[1]. Y-axis scales

represent rpm (reads per million). Representative of n = 3 independent replicates. **e,** Overlap of all annotated ORFs with ORFs with Ribo-seq coverage (TE ≥1; total RNA RPKM ≥30) and detected TIS(Ret) peaks.

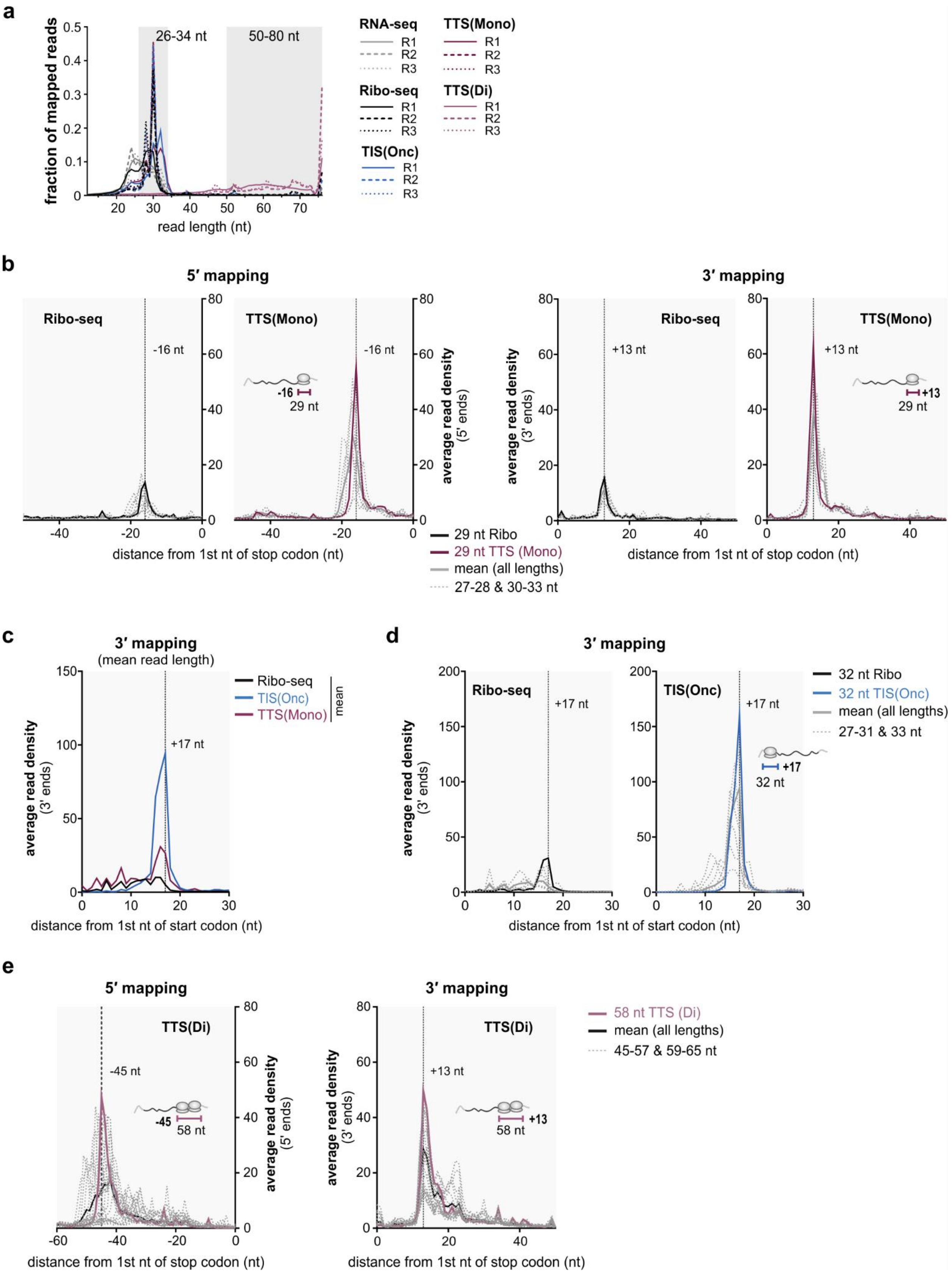
a
fraction of mapped reads
0.5
0.4
0.3
0.2
0.1
0
20
30
40
50
60
70
read length (nt)
26-34 nt
50-80 nt
RNA-seq
R1
R2
R3
Ribo-seq
R1
R2
R3
TIS(Onc)
R1
R2
R3
TTS(Mono)
R1
R2
R3
TTS(Di)
R1
R2
R3
b
5′ mapping
3′ mapping
Ribo-seq
TTS(Mono)
-16 nt
+13 nt
-16
+13
29 nt
average read density (5' ends)
average read density (3' ends)
distance from 1st nt of stop codon (nt)
29 nt Ribo
29 nt TTS (Mono)
mean (all lengths)
27-28 & 30-33 nt
c
3′ mapping
(mean read length)
Ribo-seq
TIS(Onc)
TTS(Mono)
mean
+17 nt
average read density (3' ends)
distance from 1st nt of start codon (nt)
d
3′ mapping
Ribo-seq
TIS(Onc)
+17 nt
+17
32 nt
32 nt Ribo
32 nt TIS(Onc)
mean (all lengths)
27-31 & 33 nt
average read density (3' ends)
distance from 1st nt of start codon (nt)
e
5′ mapping
3′ mapping
TTS(Di)
-45 nt
+13 nt
-45
+13
58 nt
average read density (5' ends)
average read density (3' ends)
distance from 1st nt of stop codon (nt)
58 nt TTS (Di)
mean (all lengths)
45-57 & 59-65 nt

Supplementary Figure 4. Metagene analysis of ribosome occupancy for offset and read length determination for the TTS profiling experiment. a, Read length distribution for libraries from the TIS(Onc)/TTS dataset. The size-selected RNA lengths of 26-34 nt (monosome footprints) and 50-80 nt (disome footprints) are highlighted with grey boxes. Solid line: replicate 1; dashed line: replicate 2; dotted line: replicate 3. **b,** Ribosome occupancy near stop codons using 5′/3′ read end coverage for Ribo-seq vs. TTS monosome libraries (TTS(Mono)). Solid black lines: 29 nt reads (Ribo-seq). Solid red lines: 29 nt reads (TTS(Mono)). Solid grey lines: mean of all read lengths. Dashed grey lines: all other read lengths (27-28 and 30-33 nt). Related to main **Fig. 3c**. **c,** Ribosome occupancy near start codons using 3′ read end coverage (mean of all read lengths) for the Ribo-seq, TIS(Onc), and TTS(Mono) libraries. **d,** Ribosome occupancy near start codons using 3′ read end coverage for different read lengths in Ribo-seq vs. TIS(Onc) libraries. Solid black line: 32 nt reads of Ribo-seq. Solid blue line: 32 nt reads of TIS(Onc). Solid grey lines: mean of all read lengths. Dashed grey lines: all other read lengths (27-31 and 33 nt). **e,** Ribosome occupancy near stop codons for different read lengths in the TTS disome library (TTS(Di)). Solid black lines: mean of all lengths. Solid red lines: 58 nt reads of TTS(Di). Dashed grey lines: all other read lengths (45-57 and 59-65 nt). Related to main **Fig. 3d**. Representative of *n* = 3 independent replicates. Source data are provided as a Source Data file.

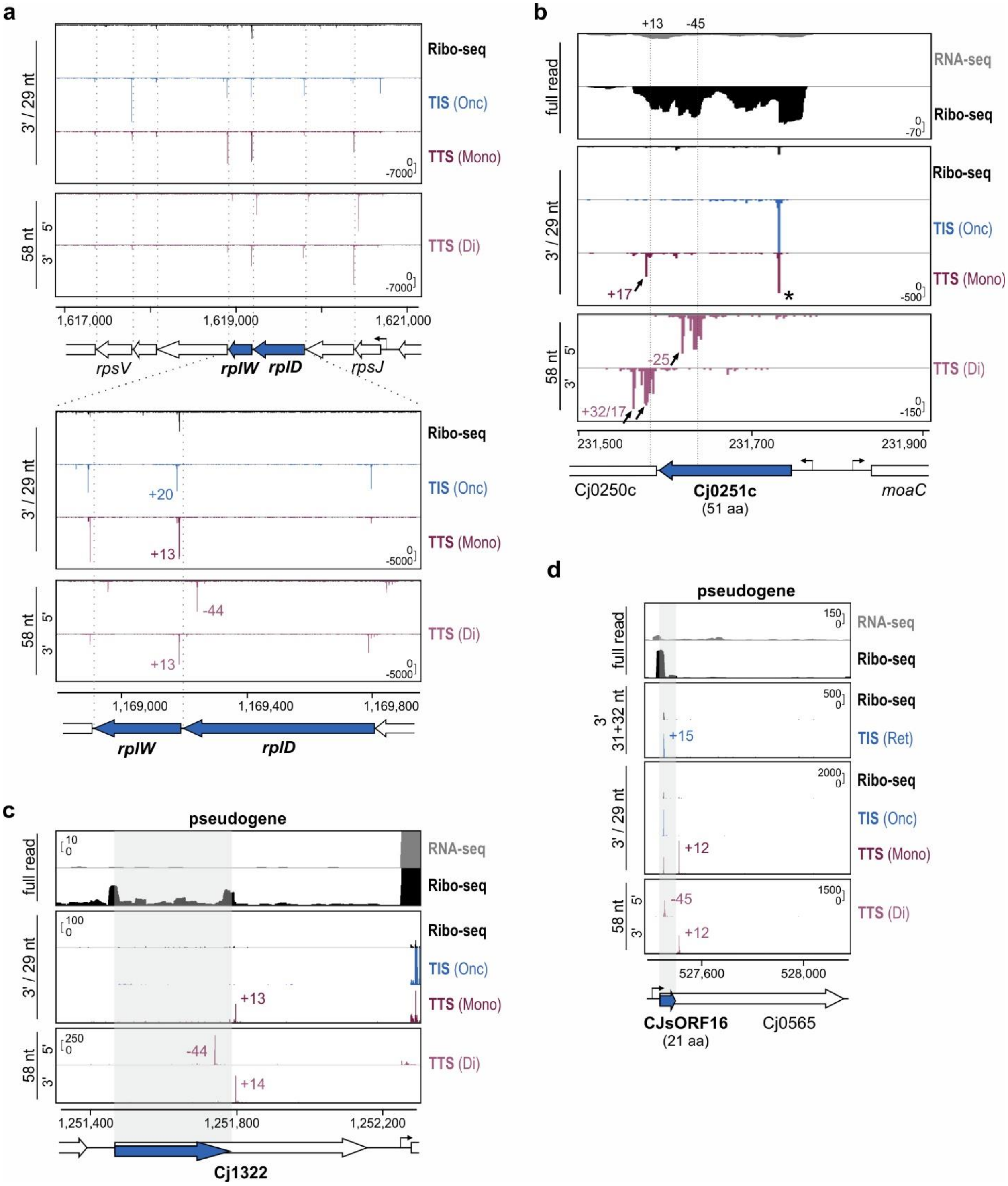

Supplementary Figure 5. Api-mediated TTS profiling reveals *C. jejuni* stop codons. a, Single-nt (read 5′ or 3′ end) mapping coverage from the TTS experiment for an r-protein operon. Bottom: zoom-in on *rplD* ORF boundaries shows peaks at the expected offsets: +13 nt for TTS(Mono) and TTS(Di) (3′ end mapping); -44 nt for TTS(Di) (5′ end mapping). The indicated TIS peak at +20 nt (vs. the *rplD* stop codon) is at the expected +17 position for the *rplW* start codon. Dashed lines: stop codon

positions. **b,** TTS coverage for the Cj0251c sORF (51 aa) demonstrates stop codon readthrough induced by Api (arrows). Asterisk: partial enrichment at the start codon by Api. Dashed lines: expected TTS positions (+13 nt (monosomes and disomes, 3′ end) and -45 nt (disome 5′ ends)). **c,** Translation of the Cj1322 pseudogene until an in-frame stop codon mutation in the reference genome. **d,** TIS/TTS-based detection of a novel sORF (CJsORF16, 21 aa) generated from translation of a pseudogene (Cj0565) until an in-frame stop codon generated by mutation. For all screenshots, bent arrows: TSS based on dRNA-seq[1]. Grey shading: Translated region of ORFs. Y-axis scales represent rpm (reads per million). Representative of *n* = 3 independent replicates.

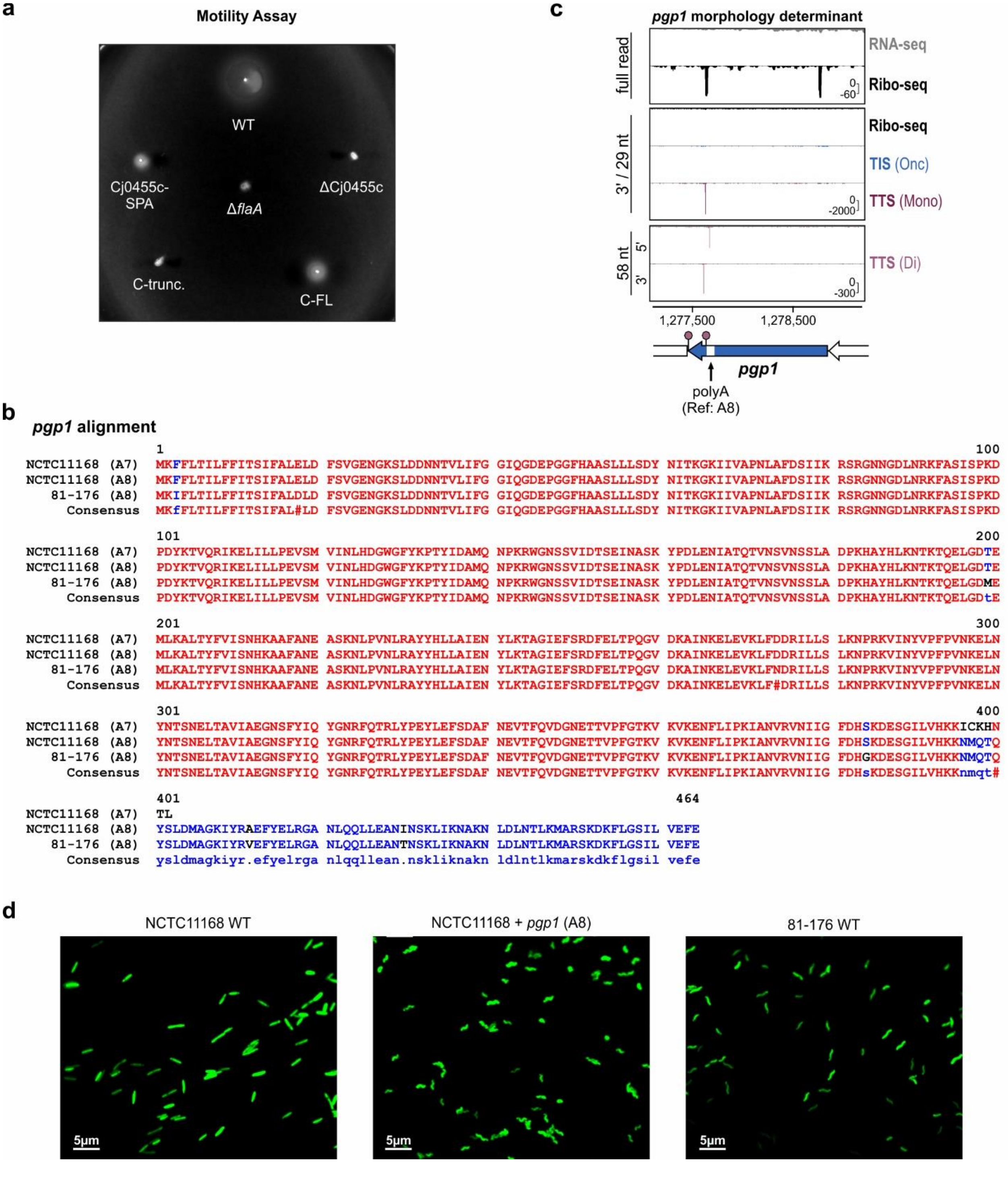

Supplementary Figure 6. Translation status of motility and shape-determining proteins. a, Representative motility assay of a *C. jejuni* ΔCj0455c mutant complemented in the chromosome either with the full-length (C-FL; CAA) or the truncated (C-trunc.; TAA) Cj0455c isoform at unrelated *rdxA*. The C-terminal SPA-tagged Cj0455c strain is included. Δ*flaA*: non-motile control. Related to main **Fig. 4b**. Representative of *n* = 3 independent experiments. **b,** Alignment of *pgp1* aa sequences from *C. jejuni* NCTC11168 (A8 reference and actual A7 determined by Sanger sequencing) and 81-

176 using MultAlin[5]. Red: 100% aa identity. Blue: >50% aa identity. **c,** TTS peaks are consistent with rod shape and *pgp1* genotype (*pgp1*-ON/OFF(A8/A7)) of the NCTC11168 WT isolate used for Ribo-seq/TTS. Representative of *n* = 3 independent replicates. **d,** Confocal micrographs for analysis of cell morphology of *C. jejuni* strains expressing *pgp1* alleles. Bacteria were stained with FITC (fluorescein isothiocyanate). Related to main **Fig. 4d**. NCTC11168 WT: straight isolate used in this study. NCTC11168 + *pgp1* (A8): WT carrying, in addition to the *pgp1* A7 allele at the native locus, a copy of *pgp1*-A8 from 81-176 at unrelated *rdxA*. 81-176 WT: WT (spiral) isolate. Representative of *n* = 2 independent replicates. Source data are provided as a Source Data file.

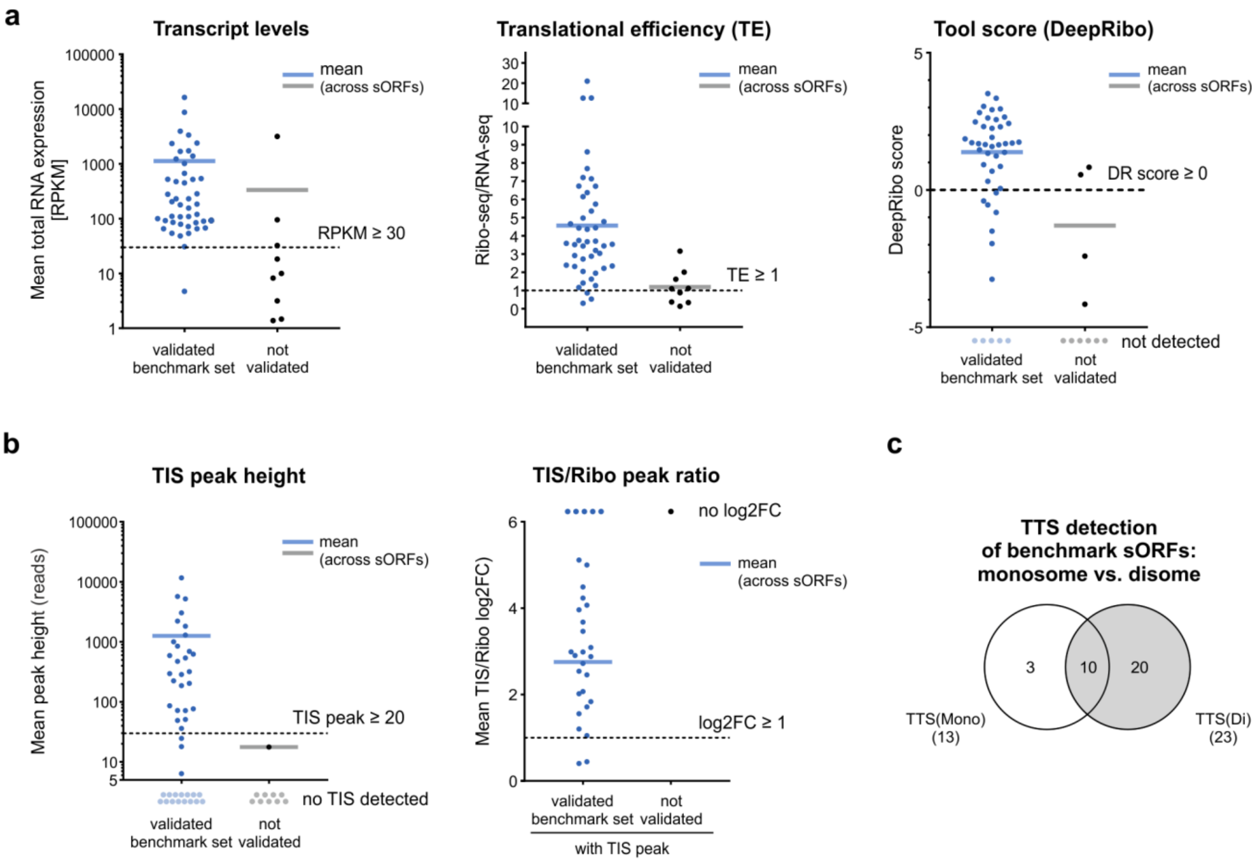

d

	**Ribo-seq**<br>Cm / no drug	**TIS profiling**<br>Ret / Onc	**TTS profiling**<br>Api (Mono / Di)
**Filtering criteria**	• ≤ 71 codons<br>• avg. TE ≥ 1, avg. RNA rpkm ≥ 30<br>• predicted in Reperation<br>• or DeepRibo (DR score>0)<br>• in minimum two independent experiments	• ≥ 11 / ≤ 71 codons<br>• peak height ≥ 20<br>• no Ribo peak or log2FC ≥ 1<br>• in minimum two replicates<br>• add top 40 candidates < 10 codons	• shortest ORF ≤ 71 codons<br>• longest ORF ≥ 11 codons<br>• no TIS(Onc) peak allowed<br>• excl. internal out of frame and within 25 nt downstream of annotated stop codon
	**manual curation**		

Supplementary Figure 7. Establishing cutoffs for novel CJsORF predictions from Ribo-seq data. **a,** Transcript levels (RPKM, *left*), translational efficiency (TE, *middle*), and DeepRibo score (*right*) for the validated (based on MS and tagging and western blot analysis) sORF benchmark set (blue) vs. not validated sORFs (black). Dashed lines: cutoffs used for predictions. Solid lines: Mean within each sORF set. Ribo-seq(Cm) is shown as a representative experiment. For DeepRibo scores, higher values indicate higher confidence predictions[6]. sORFs without predictions are indicated below (light blue/grey dots, “not detected”). **b,** TIS peak height (reads, *left*) and corresponding TIS/Ribo peak ratio (log2FC, *right*) for validated sORF benchmark set (blue) and not validated sORFs (black). Dashed lines: cutoffs for sORF predictions. Solid lines: Mean within each sORF set. TIS(Ret) was used as a representative experiment. sORFs without a TIS peak (left graph) are indicated below (light blue/grey dots). Those without a Ribo peak (TIS peak only, “no log2FC”, right graph) are shown above (blue/grey dots). sORFs without a TIS peak were not considered in the right plot. **c,** Comparison of

TTS predictions from TTS(Mono) and TTS(Di) data for the sORF benchmark set. **d,** Overview of filtering criteria for sORF candidates from all datasets. Related to main **Fig. 5a**. Source data are provided as a Source Data file.

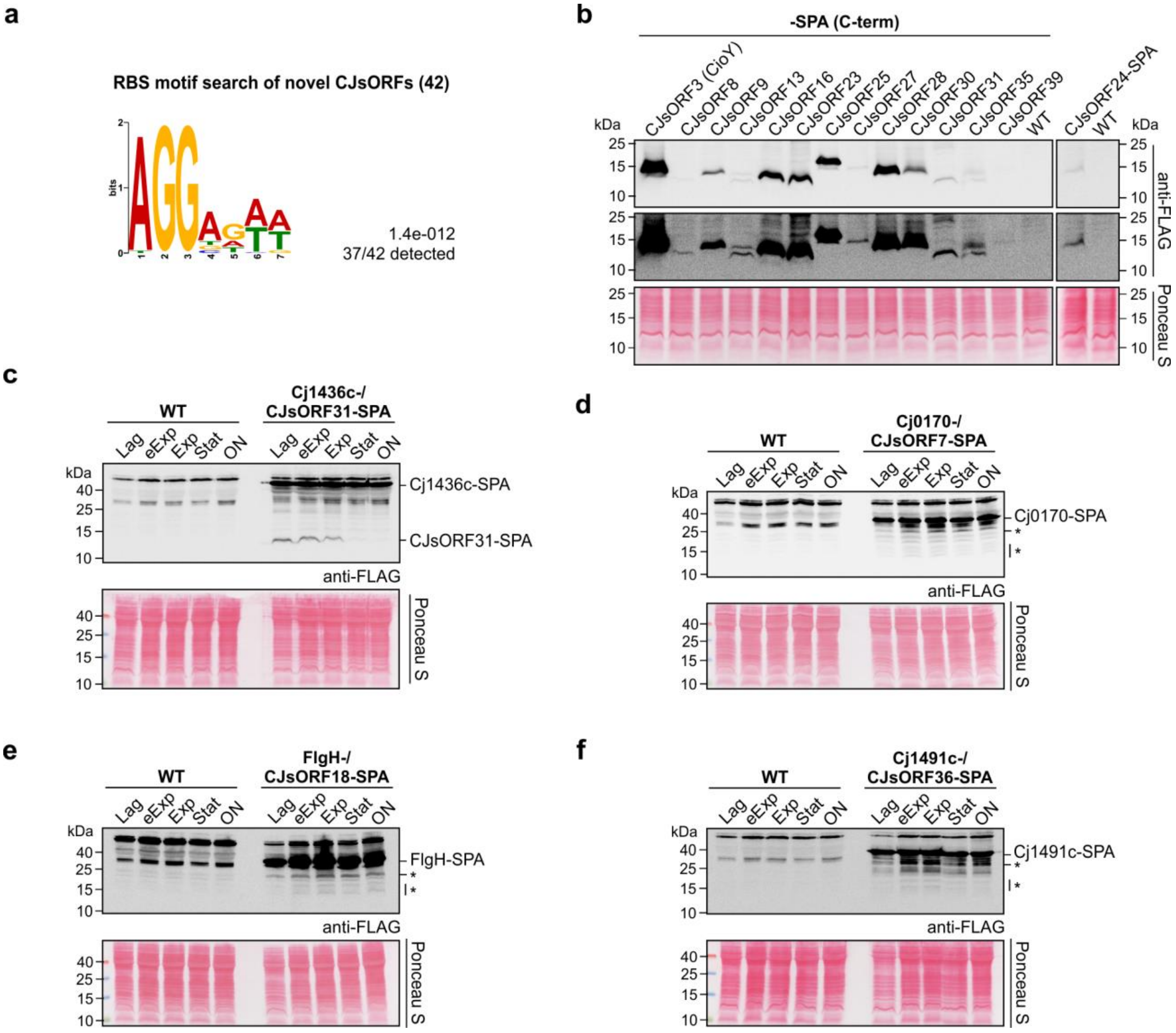

Supplementary Figure 8. Additional validation of novel CJsORFs. a, MEME analysis of 15 nt upstream of novel CJsORFs (https://meme-suite.org/meme/[7]). **b,** Western blot analysis of all detected novel CJsORFs. Samples were harvested from lag phase (at OD_{600} 0.1) from strains carrying a C-terminal SPA tag at the native locus. Middle panel: higher exposure of top panel. The CJsORF24-SPA sample, together with the WT control sample, was loaded on a separate blot from the others due to space but was processed in parallel. These specific blots were prepared one time, but all CJsORFs shown were previously validated in two independent replicates (see main **Fig. 5b**, same samples used). **c,** Full western blot for CJsORF31 (related to main **Fig. 5b**). **d-f,** Full western blot for internal in-frame candidates CJsORF7, CJsORF18, and CJsORF36. Parental genes are indicated, as introduction of the epitope at the sORF C-terminus also tags the parental gene. Asterisks: potential CJsORF bands. eExp: early exponential. Stat: Stationary. ON: overnight. For western blot, SPA-tagged proteins were detected from 0.2 OD_{600} equivalents of cell lysate with an anti-FLAG antibody. WT: untagged WT antibody negative control. Ponceau S staining of membranes served as a loading control. Western blots are representative of n = 2 independent experiments. Source data are provided as a Source Data file.

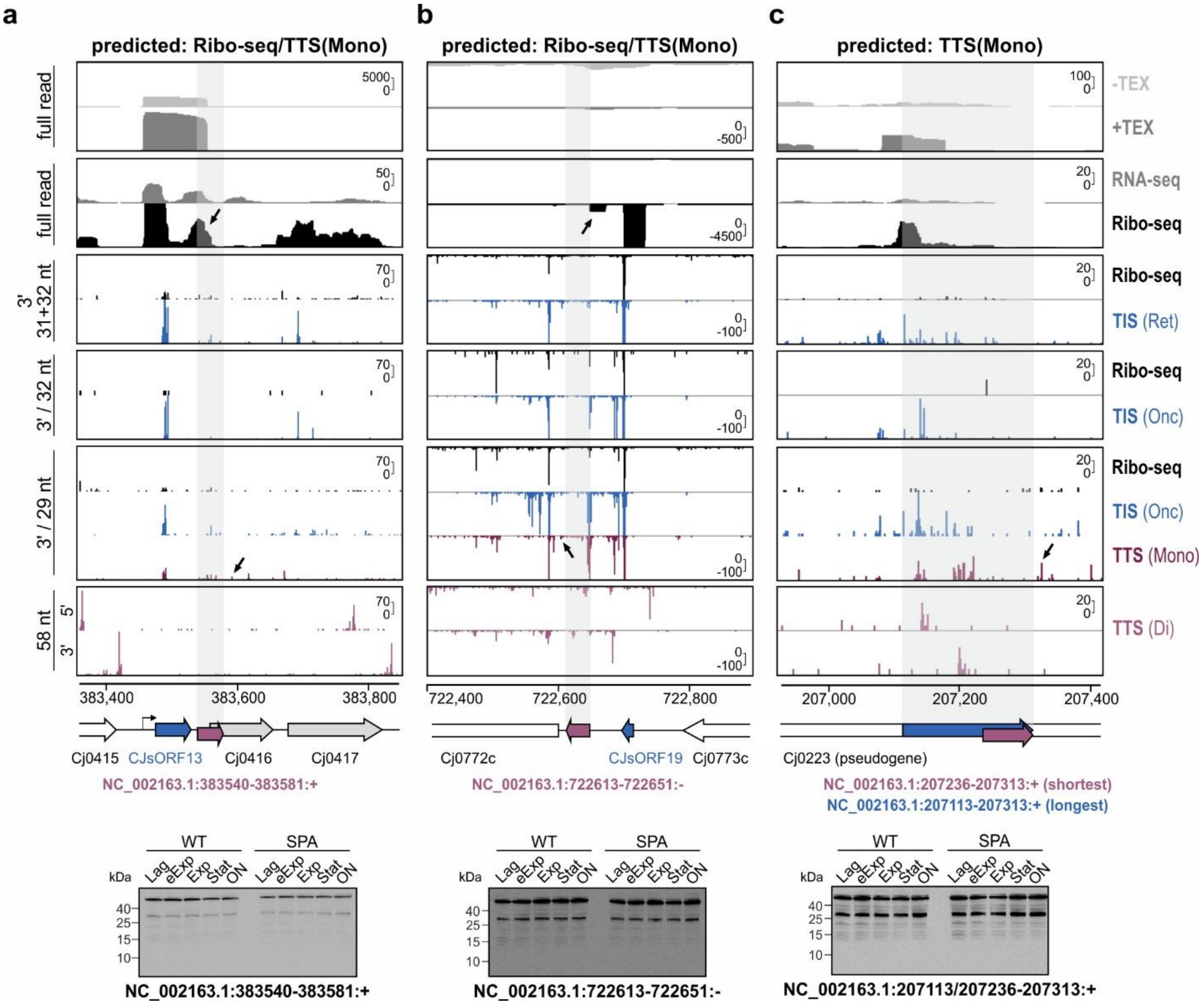

Supplementary Figure 9. Examples of sORF predictions that were discarded during manual curation and could not be detected by western blotting. a, *Top*: cDNA coverage for sORF prediction NC_002163.1:383540-383581:+ (purple arrow and label) from Ribo-seq and TTS(Mono) libraries. Bottom: Western blot analysis of C-terminal SPA tagged ORF. **b,** NC_002163.1:722613-722651:- sORF prediction (purple arrow and label) from Ribo-seq and TTS(Mono) libraries. **c,** Based on TTS(Mono) peak predicted sORFs NC_002163.1:207236-207313:+ (shortest possible sORF indicated in purple) and NC_002163.1:207113-207313:+ (longest possible sORF indicated in blue). Black arrows: coverage/peaks that led to the prediction. Grey shading indicates the position of the respective sORF prediction. Western blots are representative of n = 2 independent experiments, cDNA coverage is representative of n = 3. Source data are provided as a Source Data file.

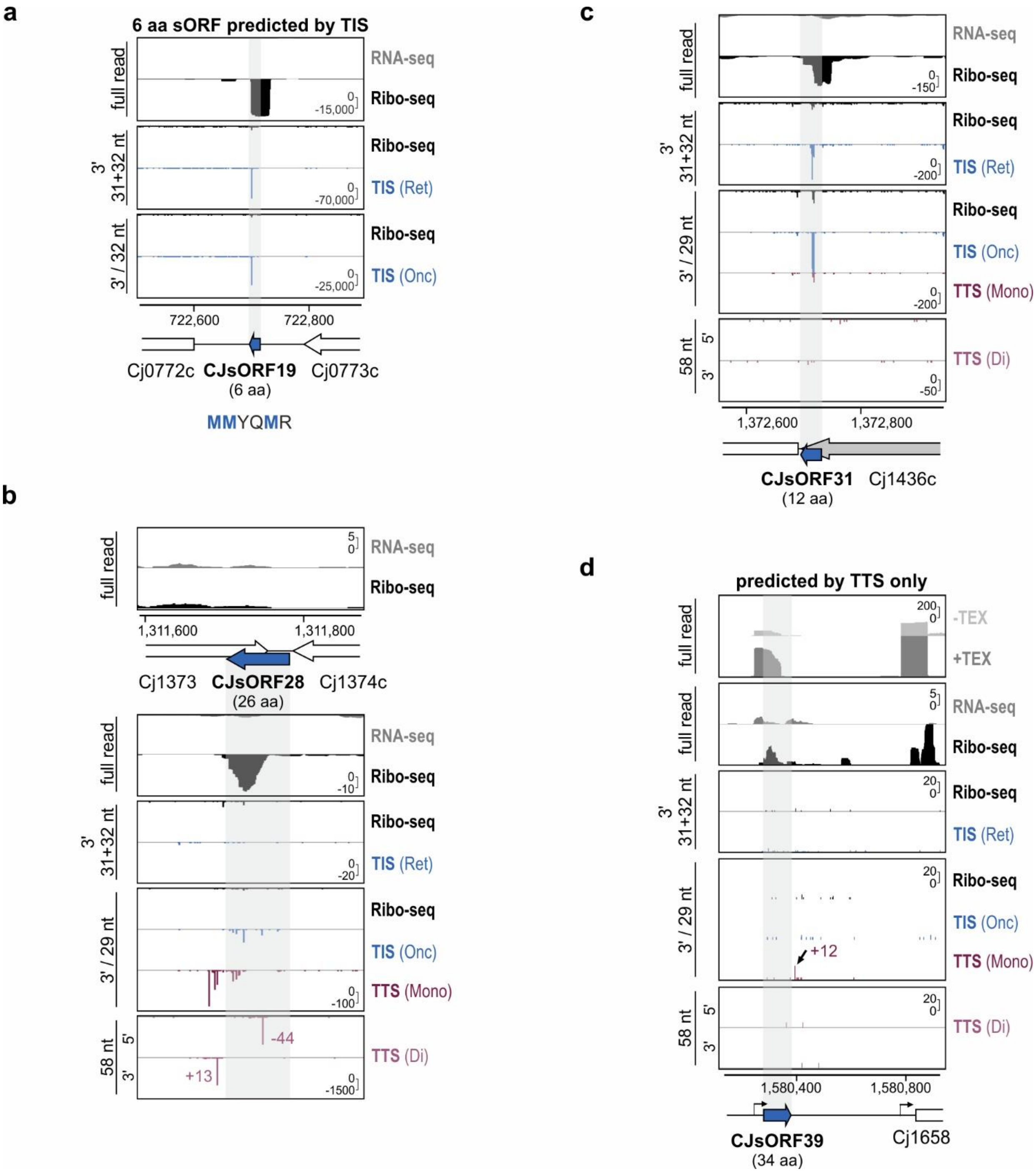

Supplementary Figure 10. Novel CJsORFs predicted from TIS (Ret and Onc) and TTS profiling data. a, Short ORF CJsORF19 (6 aa) was a top TIS candidate < 10 codons, is encoded next to methionine transport genes, and is enriched for methionine codons. **b, c** Full coverage for novel sORFs CJsORF28 and CJsORF31. Related to main **Fig. 5e**. **d,** Intergenic CJsORF39 was predicted from TTS data alone. Bent arrows: TSS based on dRNA-seq[1]. Y-axis: rpm (reads per million). Grey shading:Position of sORFs. Representative of *n* = 3 independent replicates.

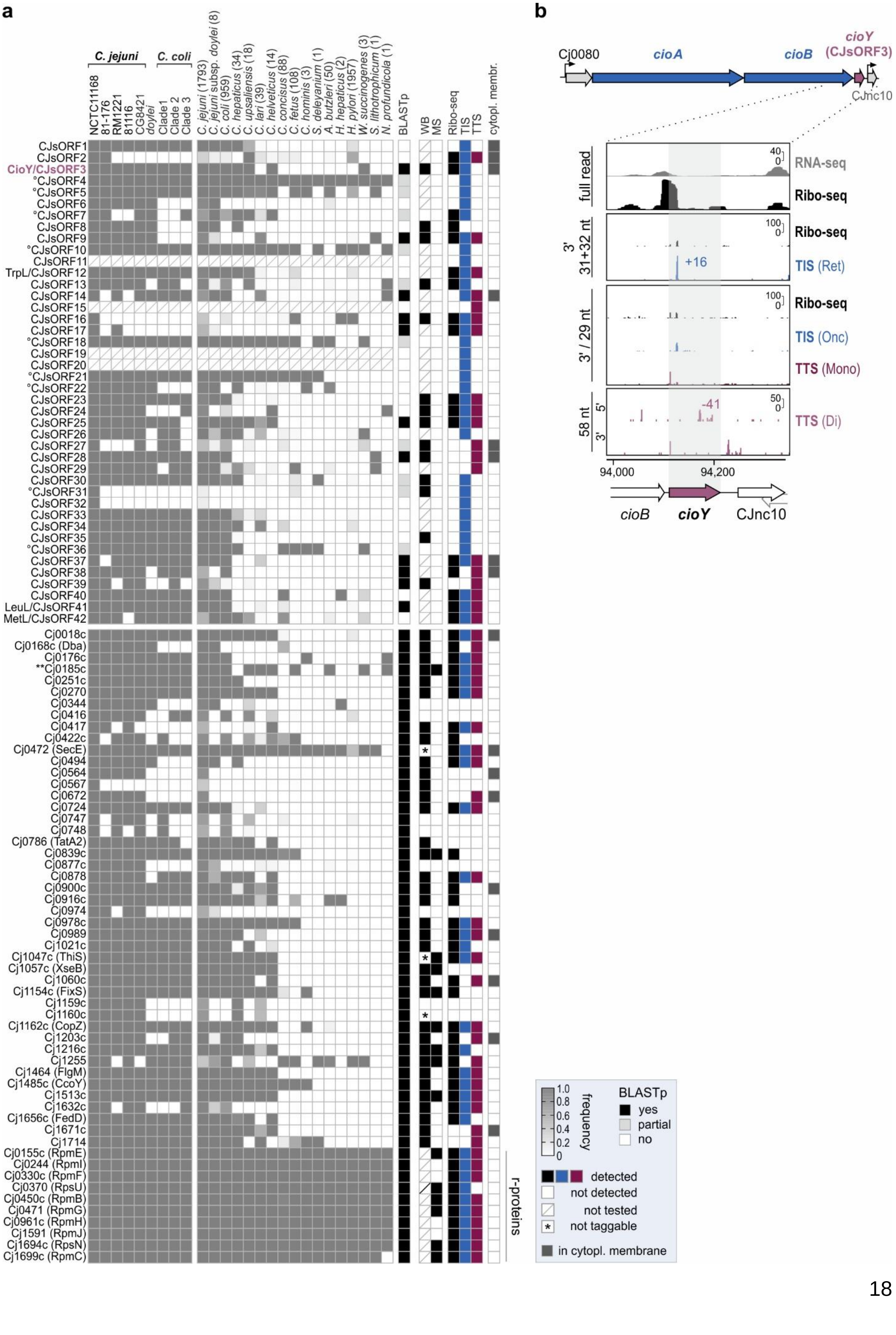
a
b
C. jejuni
C. coli
NCTC11168
81-176
RM1221
81116
CG8421
doylei
Clade1
Clade 2
Clade 3
BLASTp
WB
MS
Ribo-seq
TIS
TTS
cytopl. membr.
r-proteins
CioY/CJsORF3
TrpL/CJsORF12
LeuL/CJsORF41
MetL/CJsORF42
Cj0472 (SecE)
Cj0786 (TatA2)
Cj1047c (ThiS)
Cj1057c (XseB)
Cj1154c (FixS)
Cj1162c (CopZ)
Cj1464 (FlgM)
Cj1485c (CcoY)
Cj1656c (FedD)
Cj0155c (RpmE)
Cj0244 (RpmI)
Cj0330c (RpmF)
Cj0370 (RpsU)
Cj0450c (RpmB)
Cj0471 (RpmG)
Cj0961c (RpmH)
Cj1591 (RpmJ)
Cj1694c (RpsN)
Cj1699c (RpmC)
frequency
BLASTp
yes
partial
no
detected
not detected
not tested
not taggable
in cytopl. membrane
Cj0080
cioA
cioB
cioY
(CJsORF3)
CJnc10
full read
RNA-seq
Ribo-seq
31+32 nt
+16
TIS (Ret)
3' / 29 nt
TIS (Onc)
TTS (Mono)
58 nt
-41
TTS (Di)
94,000
94,200

Supplementary Figure 11. Conservation of novel sORF candidates in Epsilonproteobacteria reveals a terminal oxidase small protein component *cioY*. a, Extended conservation analysis of novel *C. jejuni* small proteins. Related to main **Fig. 6a**. (°): internal in-frame candidates. Western Blot (WB): Epitope-tagged version detected by western blot at any growth phase. MS: detected by mass spectrometry in log phase. Cytoplasmic membrane (Cytopl. memb.) localisation was predicted by pSORTb[8]. Conservation (centre/grey): the frequency of detection, using the NCTC11168 homolog as query, within the indicated number of high quality genomes (in brackets) is shown based on tblastn (see Methods for details of genome quality control and tblastn analysis). BLASTp: yes - at least one 100% match in length and sequence. partial - sequence aligns with part of an (often longer) annotated ORF (see also **Supplementary Data file 5**). **b,** Translatomics coverage (representative of n = 3) for *cioY* (CJsORF3, 34 aa) located in the conserved Cj0080-*cioA*-*cioB*-*cioY* operon. Bent arrows: TSS based on dRNA-seq[1]. Y-axis: rpm (reads per million).

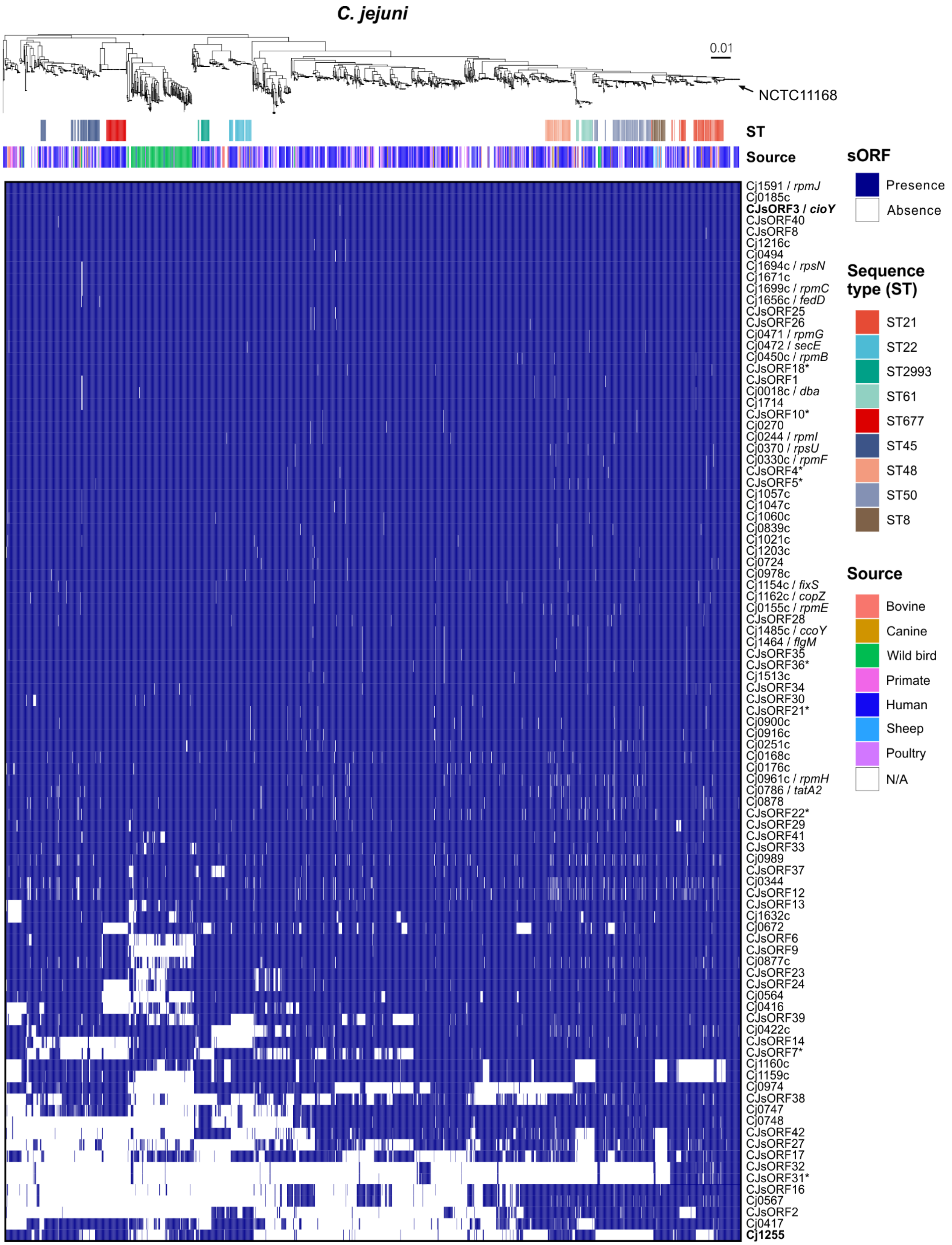

C. jejuni
0.01
NCTC11168
ST
Source
sORF
Presence
Absence
Sequence type (ST)
ST21
ST22
ST2993
ST61
ST677
ST45
ST48
ST50
ST8
Source
Bovine
Canine
Wild bird
Primate
Human
Sheep
Poultry
N/A
Cj1591 / rpmJ
Cj0185c
CJsORF3 / cioY
CJsORF40
CJsORF8
Cj1216c
Cj0494
Cj1694c / rpsN
Cj1671c
Cj1699c / rpmC
Cj1656c / fedD
CJsORF25
CJsORF26
Cj0471 / rpmG
Cj0472 / secE
Cj0450c / rpmB
CJsORF18*
CJsORF1
Cj0018c / dba
Cj1714
CJsORF10*
Cj0270
Cj0244 / rpmI
Cj0370 / rpsU
Cj0330c / rpmF
CJsORF4*
CJsORF5*
Cj1057c
Cj1047c
Cj1060c
Cj0839c
Cj1021c
Cj1203c
Cj0724
Cj0978c
Cj1154c / fixS
Cj1162c / copZ
Cj0155c / rpmE
CJsORF28
Cj1485c / ccoY
Cj1464 / flgM
CJsORF35
CJsORF36*
Cj1513c
CJsORF34
CJsORF30
CJsORF21*
Cj0900c
Cj0916c
Cj0251c
Cj0168c
Cj0176c
Cj0961c / rpmH
Cj0786 / tatA2
Cj0878
CJsORF22*
CJsORF29
CJsORF41
CJsORF33
Cj0989
CJsORF37
Cj0344
CJsORF12
CJsORF13
Cj1632c
Cj0672
CJsORF6
CJsORF9
Cj0877c
CJsORF23
CJsORF24
Cj0564
Cj0416
CJsORF39
Cj0422c
CJsORF14
CJsORF7*
Cj1160c
Cj1159c
Cj0974
CJsORF38
Cj0747
Cj0748
CJsORF42
CJsORF27
CJsORF17
CJsORF32
CJsORF31*
CJsORF16
Cj0567
CJsORF2
Cj0417
Cj1255

Supplementary Figure 12. Detailed sORF conservation (presence/absence) in *C. jejuni* strains. The *C. jejuni* NCTC11168 sORF sequence was used for tblastn searches against a custom database of high quality sequences (n=1793) from NCBI (**Supplementary Data file 8**). To be considered detected, we applied a cutoff of 50% identity and 50% coverage, with an E-value <1. Top: phylogenetic tree based on core genome SNPs. (*): Internal In-frame candidates.

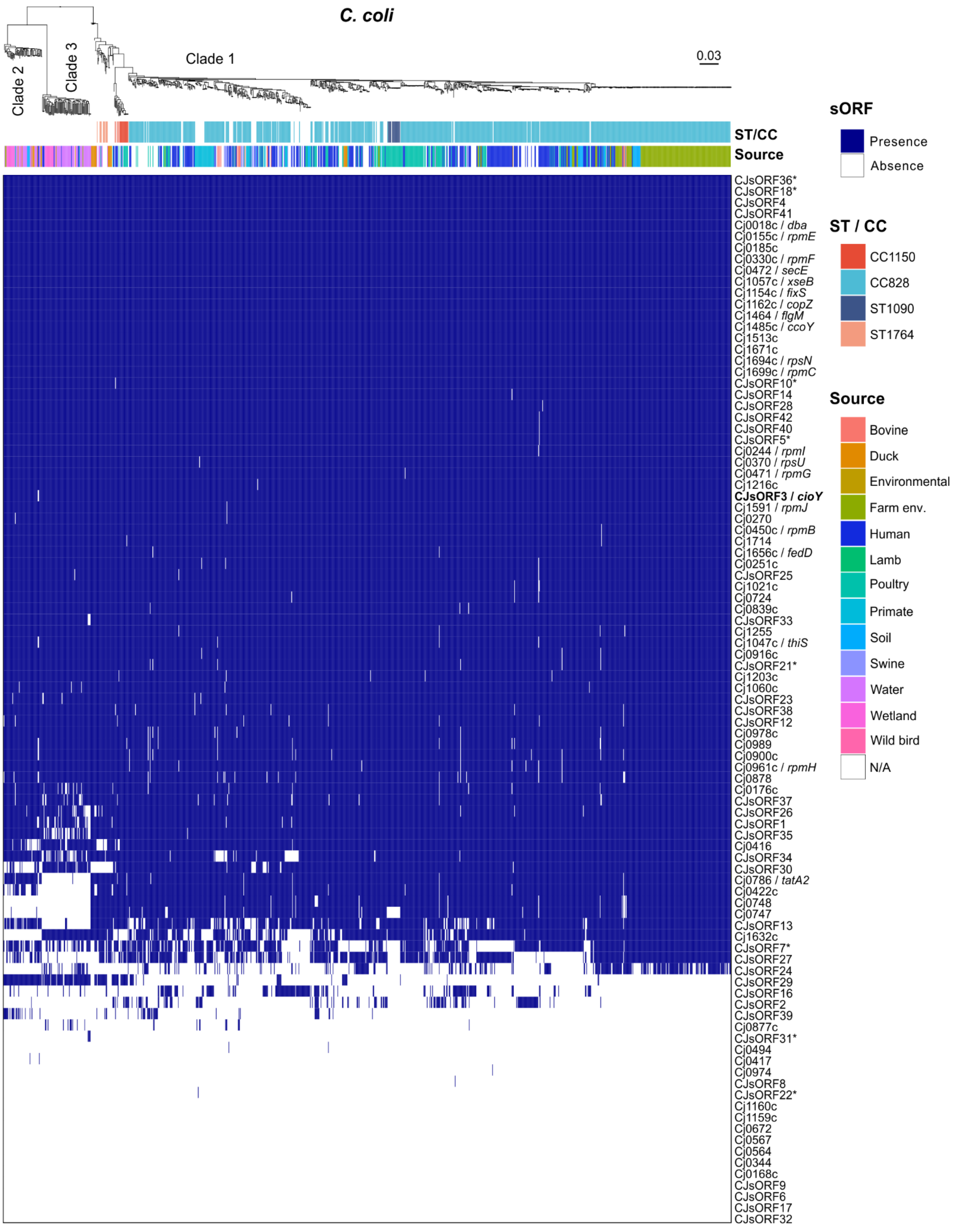
C. coli
Clade 2
Clade 3
Clade 1
0.03
ST/CC
Source
sORF
Presence
Absence
ST / CC
CC1150
CC828
ST1090
ST1764
Source
Bovine
Duck
Environmental
Farm env.
Human
Lamb
Poultry
Primate
Soil
Swine
Water
Wetland
Wild bird
N/A
CJsORF36*
CJsORF18*
CJsORF4
CJsORF41
Cj0018c / dba
Cj0155c / rpmE
Cj0185c
Cj0330c / rpmF
Cj0472 / secE
Cj1057c / xseB
Cj1154c / fixS
Cj1162c / copZ
Cj1464 / flgM
Cj1485c / ccoY
Cj1513c
Cj1671c
Cj1694c / rpsN
Cj1699c / rpmC
CJsORF10*
CJsORF14
CJsORF28
CJsORF42
CJsORF40
CJsORF5*
Cj0244 / rpmI
Cj0370 / rpsU
Cj0471 / rpmG
Cj1216c
CJsORF3 / cioY
Cj1591 / rpmJ
Cj0270
Cj0450c / rpmB
Cj1714
Cj1656c / fedD
Cj0251c
CJsORF25
Cj1021c
Cj0724
Cj0839c
CJsORF33
Cj1255
Cj1047c / thiS
Cj0916c
CJsORF21*
Cj1203c
Cj1060c
CJsORF23
CJsORF38
CJsORF12
Cj0978c
Cj0989
Cj0900c
Cj0961c / rpmH
Cj0878
Cj0176c
CJsORF37
CJsORF26
CJsORF1
CJsORF35
Cj0416
CJsORF34
CJsORF30
Cj0786 / tatA2
Cj0422c
Cj0748
Cj0747
CJsORF13
Cj1632c
CJsORF7*
CJsORF27
CJsORF24
CJsORF29
CJsORF16
CJsORF2
CJsORF39
Cj0877c
CJsORF31*
Cj0494
Cj0417
Cj0974
CJsORF8
CJsORF22*
Cj1160c
Cj1159c
Cj0672
Cj0567
Cj0564
Cj0344
Cj0168c
CJsORF9
CJsORF6
CJsORF17
CJsORF32

Supplementary Figure 13. Detailed sORF conservation (presence/absence) in *C. coli* strains. The *C. jejuni* NCTC11168 sORF sequence was used for tblastn searches against a custom database of high quality sequences (n=1089) from NCBI (**Supplementary Data file 8**). To be considered detected, we applied a cutoff of 50% identity and 50% coverage, with an E-value <1. Top: phylogenetic tree based on core genome SNPs. Clade 2 and Clade 3 were defined based on strains 76339 and H093580632. (*): Internal In-frame candidates.

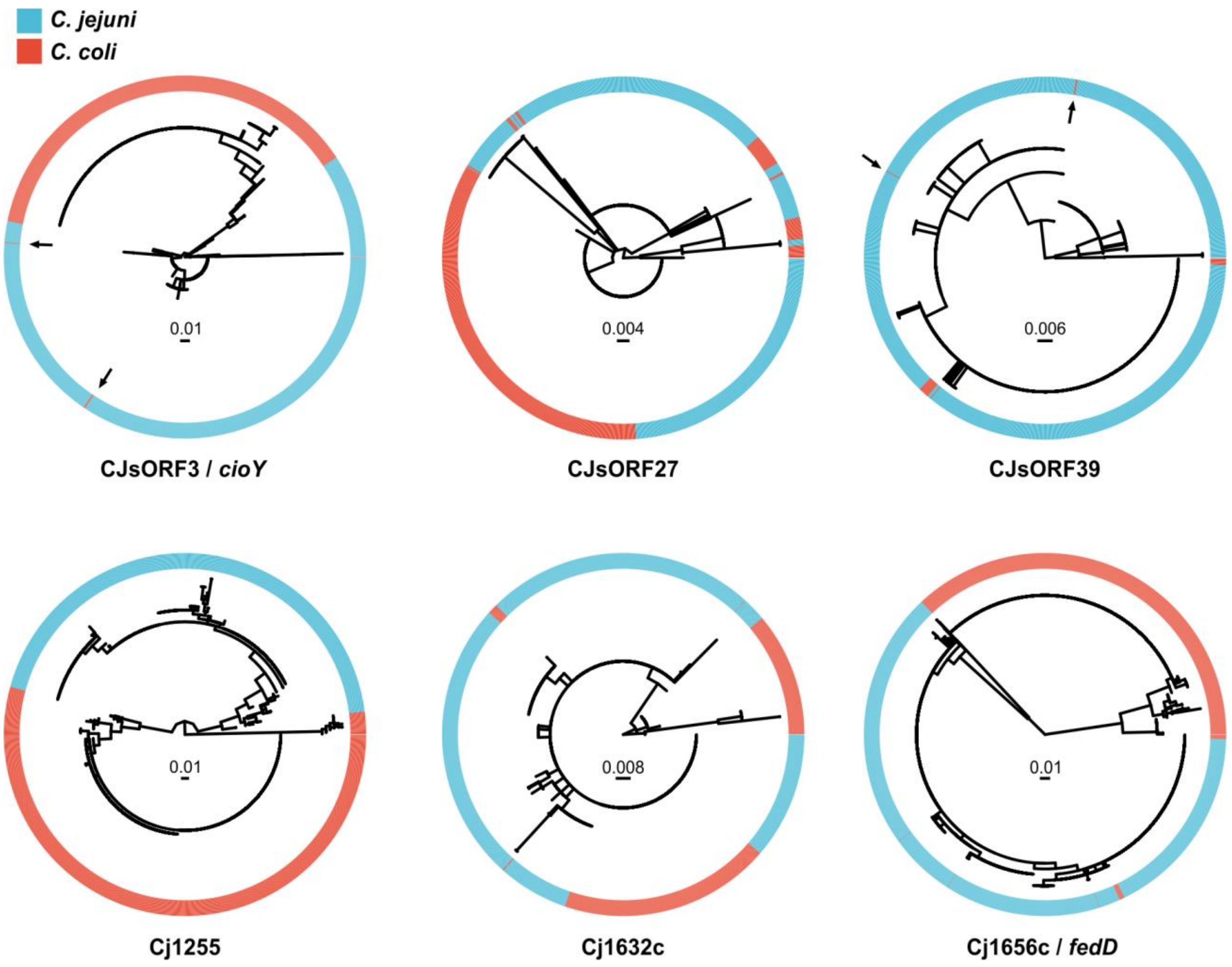

Supplementary Figure 14. Potential cross-species transfer of selected sORF candidates between *C. jejuni* and *C. coli*. Sequences of sORFs were identified in *C. jejuni* (blue) and *C. coli* (red) genomes using blastn. Extracted sequences were aligned with MAFFT version 7[9] and used to generate a phylogenetic tree with FastTree[10].

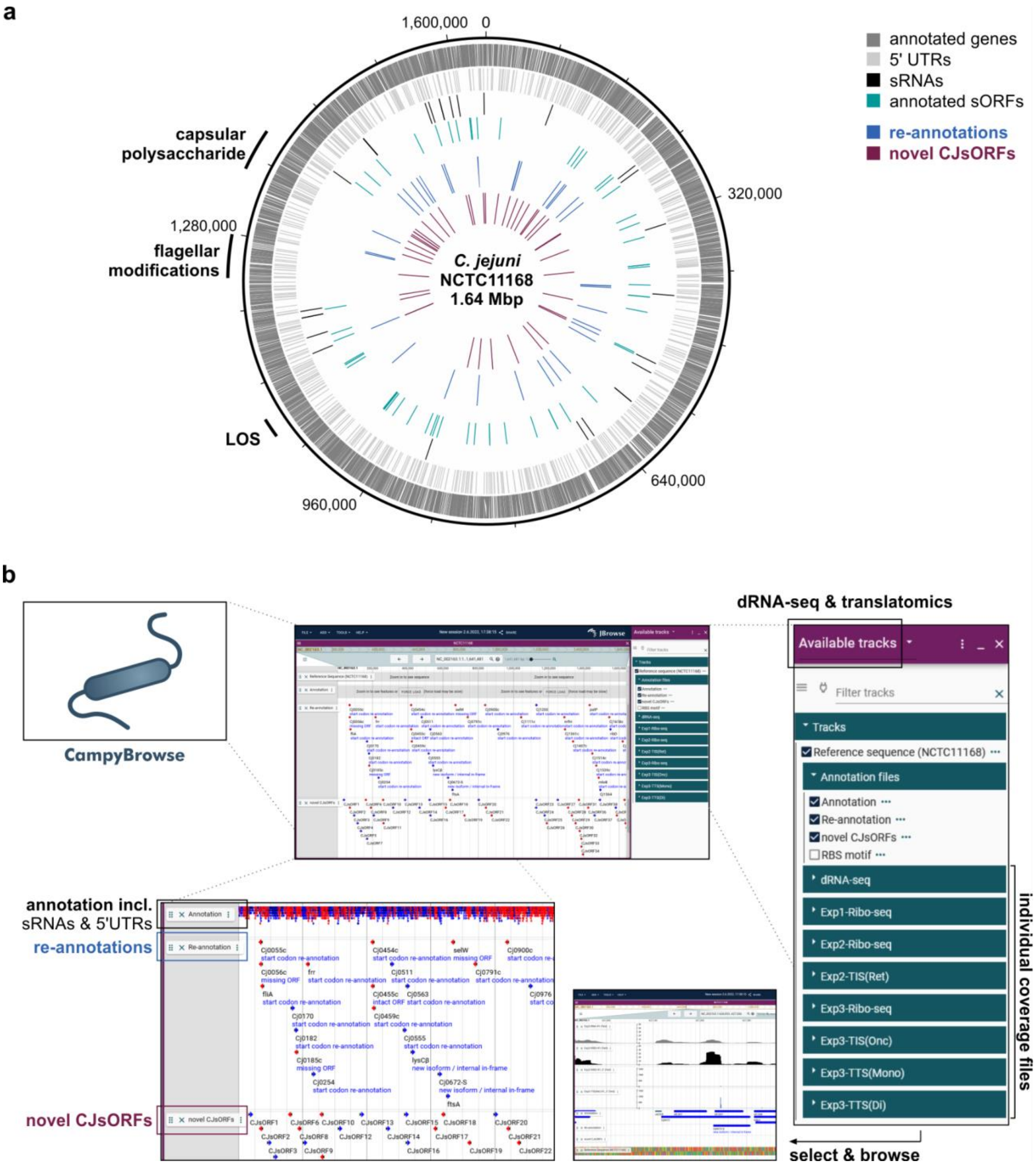

Supplementary Figure 15: CampyBrowse online resource makes *C. jejuni* NCTC11168 annotation refinements broadly accessible. a, Genome ring displaying new or updated annotated features as well as previously published 5'UTR and sRNA annotations[1]. Dark grey: NCBI annotation. Light grey/black: 5′UTRs/sRNAs[1]. Green: sORFs (≤ 70 aa) in NCBI annotation. Blue: re-annotations/additions based on this study. Red: novel CJsORFs identified in this study. Hypervariable LOS (lipooligosaccharide) biosynthesis, capsular polysaccharide biosynthesis, and flagellar

modification gene clusters are indicated[11]. Genomic features were visualized with Artemis DNA Plotter[12]. **b,** The JBrowse[13]-based *CampyBrowse* browser includes the NCBI annotation, sRNAs and 5'UTRs, dRNA-seq coverage files, as well as all translatomics-based datasets and proposed annotation updates from this study. Annotation update tracks include missing genes, identified isoforms, start/stop codon re-annotations, and the novel CJsORFs. A detailed "ReadMe" provides advice on how to use the browser as well as how to interpret the data. *CampyBrowse* is accessible via: *http://www.bioinf.uni-freiburg.de/~ribobase/campybrowse/overview.html*.

Online Methods

Transformation for *C. jejuni* mutant construction

Strains grown from frozen stocks until passage one or two on MH agar (Becton Dickinson) were harvested into cold electroporation solution (272 mM sucrose, 15% (v/v) glycerol) and washed twice with the same buffer. Cells (50 µl) were mixed with 500-1000 ng PCR product on ice and electroporated (Bio-rad MicroPulser) in a 1 mm gap cuvette at 2.5 kV. Cells were then transferred with *Brucella* broth (Becton Dickinson) to a non-selective MH plate and recovered overnight at 37°C micro-aerobically before plating on the appropriate selective medium. Strains were validated by colony PCR and for complementation, overexpression, and epitope tagging, validated by Sanger sequencing (Macrogen, Microsynth).

***C. jejuni* non-polar deletion mutant construction by recombination with overlap PCR products.** Non-polar deletion mutants of protein coding-genes were constructed by homologous recombination with overlap PCR products consisting of a resistance cassette in between approximately 500 bp of sequence upstream and downstream of the target gene using primers listed in **Supplementary Data file 11**. As an example, deletion of *cmeB* with a non-polar Gm^R cassette is described. An approximately 500 bp region upstream of *cmeB* (Cj0366c) was amplified using CSO-4091/4092, while the downstream region was amplified using CSO-4093/4094. The 5′ ends of the antisense primer for the upstream region and the sense primer for the downstream region included regions overlapping the resistance cassette 5′ and 3′ end, respectively. The Gm^R cassette was amplified using primers HPK1/HPK2 from pUC1813-apra[14]. Next, a three-fragment overlap PCR was performed using the *cmeB* upstream, *cmeB* downstream, and Gm^R cassette fragments in an equimolar ratio and primers CSO-4091/4094. Following confirmation of the correct size by gel electrophoresis, the resulting overlap PCR product was electroporated as described above into WT and deletion mutants were selected on plates containing gentamicin. The deletion strain (Δ*cmeB*, CSS-5613) was confirmed using a primer binding upstream of the *cmeB* upstream fragment (CSO-4091) and an antisense primer binding the Gm^R cassette

(HPK2). For non-polar Gm^R, Kan^R, and Hyg^R deletions, cassettes were amplified using HPK1/HPK2 from pUC1813-apra[14], pGG1[15], or pACH1[16] as template, respectively. For PCR reactions including the Hyg^R cassette, 3% DMSO was included in reactions.

C-terminal 3×FLAG-, SPA-, or sfGFP-tagging. Epitope tagging was performed via homologous recombination at the native locus. The overlap PCR product contained approximately 500 bp upstream of the sORF penultimate codon fused to the 3×FLAG, SPA, or sfGFP sequence, a Kan^R or Gm^R resistance cassette, and the ORF downstream region. As an example, tagging of Cj0978c with 3×FLAG is provided. The Cj0978c upstream and coding region was amplified using CSO-3344/3770, and 500 bp of the downstream was amplified with CSO-3346/3347. A fusion of 3×FLAG to the Kan^R cassette was amplified using CSO-0065/HPK2 on the previously published 3×FLAG strain (*csrA*::3×FLAG)[15]. The up, down, and 3×FLAG-Kan^R fragments were mixed together in approximately equimolar ratios, annealed, and amplified by overlap PCR using primers CSO-3344/3347. Clones were validated by colony PCR with CSO-3348/HPK2 and sequencing with CSO-0023 or CSO-3348.

Heterologous expression from *rdxA*. The *rdxA* locus (Cj1066) can be used for heterologous gene expression in *C. jejuni*[17]. Constructs for complementation in *rdxA* were made mostly in plasmids containing approximately 500 bp of upstream and downstream sequence from *rdxA* flanking a Cm^R or Kan^R cassette (with promoter and terminator) by subcloning the *C. jejuni* sequence into previously-constructed plasmid vectors based on pFK8.5, pSE59.1, or pGD34.7[18,19]. To generate pMA16.1 (for heterologous expression of *pgp1* from *rdxA*) the backbone of pGD34.7 was amplified with CSO-3641/0350 and two inserts were amplified from 81-176 WT (CSS-0063) gDNA with CSO-3646/3647 (promoter region) and Cjj81176_1344 (*pgp1*-A8) with CSO-3644/3645. Both insert fragments were fused via overlap PCR with CSO-3646/3645, digested with *Nde*I/*Xma*I, and ligated into the plasmid backbone. Positive clones were identified by colony PCR with CSO-0463/3270 and sequencing with CSO-0646/3270. For Cj0455c complementation, inserts for Cj0455c full-length were amplified with CSO-5851/5852 or for Cj0455c truncated with CSO-5851/5853 from WT gDNA (CSS-5295), digested with *Bam*HI and ligated into a backbone amplified

from pSE59.1 with CSO-5284/1354). Positive clones were identified by sequencing with CSO-3270. Alternatively, overlap PCR was used for, *e.g.*, CJsORF35-SPA.

The backbone for generation of pFK8.5 was amplified from pST7.2 (similar to published pST1.1[18]) with CSO-0347/4738 and digested with *Cla*I. The insert (CJnc230 sRNA,[1]) was amplified with CSO-4254/4257 from WT gDNA (CSS-5295) and also digested with *Cla*I. Vector and insert were ligated, transformed into *E. coli* TOP10 cells and validated by colony PCR with CSO-2276/4257. Next, a *rdxA*_UP-Kan^R-P_{porA} fragment was amplified with CSO-2276/4738, the *rdxA*_DN region with CSO-1785/2277 from pFK8.5, and the SPA sequence was amplified with CSNIH-0080/CSO-4891 from pJL148. The CJsORF35 insert (including 103 nt upstream) was amplified from WT gDNA (CSS-5295) with CSO-4889/4890. The four fragments were mixed in an approximately equimolar ratio and subjected to overlap-extension PCR with CSO-2276/2277. This product was used for electroporation. Primers CSO-2276/2277 were used to amplify all constructs from *rdxA*-based plasmids for electroporation into *C. jejuni*. Clones with intended insertions were validated by colony PCR using CSO-0643/0644/0349 (*rdxA*-Cm^R constructs) or CSO-0023/0349 (*rdxA*-Kan^R constructs). Insertions were sequenced with CSO-3270, CSO-0643, CSO-0644, or CSO-0023.

Growth and harvest for ribosome profiling (Cm). Translation was arrested in WT *C. jejuni* cultures and cells harvested for Ribo-seq using Cm ice as described previously with modifications[20,21] as follows. *C. jejuni* NCTC11168 WT cultures were grown to mid-log phase (OD_{600} approx. 0.5) in 100 ml BB medium at 37˚C with shaking at 150 rpm under microaerobic conditions. A sample for total RNA was transferred to RNA stop mix (95% ethanol, 5% buffer-saturated phenol (Roth)) and snap-frozen in liquid N_2. Bacteria were then treated with 1 mg/ml Cm (Sigma) for 5 min at 37°C under microaerobic conditions, followed by immediate chilling by mixture with an equal volume of crushed ice containing 1 mg/ml Cm and incubation on ice for 10 minutes. Cells were harvested by centrifugation at 10,000 *g* for 10 min, and immediately frozen in liquid N_2.

Minimum inhibitory concentration (MIC) determination for TIS/TTS profiling antibiotics. To determine the sensitivity of *C. jejuni* strains to antibiotics used for TIS/TTS profiling, we measured their MIC[22] in the same broth as used for Ribo-seq using a 96 well-

plate format with serial 2-fold dilutions of antibiotic. Briefly, strains from an overnight culture in BB with vancomycin (WT (CSS-5295), Δ*cmeB* (CSS-5617) or ΔCj0182 (CSS-4077, *sbmA* homolog)) were diluted into fresh medium to an OD_{600} of 0.0005. Antibiotics (Ret - Sigma CDS023386, Onc & Api - NovoPro BioScience Inc., Shanghai, China) were diluted serially by 2-fold in BB with vancomycin, starting at a final concentration (including bacterial suspension) of 16 µg/ml for tetracycline (in ethanol, Carl Roth Art. No. 0237.2) or Ret (in dimethyl sulfoxide), or 100 µM PrAMPs. After addition of bacteria to the wells, plates were incubated under microaerobic conditions at 37°C for 24 hours. The MIC was taken as the lowest concentration where no visible bacterial growth was observed.

	WT	Δ*cmeB*	ΔCj0182
Tetracycline (µg/ml)	0.0625	0.031	0.031
Retapamulin (µg/ml)	0.125	0.016	N/A
Oncocin (µM)	0.78-1.56	N/A	100
Apidaecin-137 (µM)	3.125-6.25	N/A	>100

Growth and harvest for TIS profiling (Ret). *C. jejuni* Δ*cmeB* was grown, treated, and harvested for TIS profiling as described for Cm Ribo-seq above with minor modifications based on protocols established previously in *E. coli*[23,24] as follows. Two cultures were grown in parallel until exponential growth phase in BB medium, and a sample was removed, mixed with RNA stop mix, and immediately frozen in liquid N_2 for total RNA analysis. One culture was then treated with Ret (12.5 µg/ml final concentration) for 10 minutes under routine growth conditions. Cells were then harvested by fast-filtration with a 0.45 µm polyethersulfone membrane (Millipore) and immediately frozen in liquid N_2.

Growth and harvest for TIS profiling (Onc) and TTS. For the TIS/TTS(Onc/Api) experiment, *C. jejuni* WT was grown in BB medium until log phase (OD_{600} 0.4) in three parallel cultures. A sample was removed for total RNA analysis, mixed with RNA stop mix, and immediately frozen in liquid N_2. One culture was left untreated, while one was treated

with Onc or Api (50 µM final concentration) for 10 min under routine growth conditions, respectively. Cultures were then immediately transferred to pre-chilled glass flasks and swirled in an ice bath for 3 min to rapidly chill the cells and halt translation. Chilled cells were harvested by centrifugation at 4500 rpm and immediately frozen in liquid N_2.

RNA isolation. RNA was extracted from sucrose gradient fractions using hot phenol-chloroform-isoamyl alcohol (PCI) as performed as follows. Samples were thawed and sodium dodecyl sulfate (SDS) was added to a final concentration of 1%. After heating at 64°C for 2 minutes, 1 volume of pre-warmed PCI (Roth) was added, and samples were incubated for another 5 minutes with periodic mixing. After incubation on ice for 5 minutes, phases were separated by centrifugation at 13,000 rpm, for 5 minutes at 4°C. The aqueous phase was extracted again with an equal volume of PCI at room temperature. Following centrifugation at 13,000 rpm, for 5 minutes at 4°C , RNA was precipitated with 1/10 volume of 3 M sodium acetate, pH 5.5, 0.5 µl Glycoblue (Ambion) and 1.5 volumes of isopropanol overnight at -20°C. RNA was collected by centrifugation at 13,000 rpm at 4°C for 30 minutes, washed once with cold 75% ethanol, dried, and resuspended with RNase-free water.

Total RNA was isolated from cell pellets using the hot phenol method. Cell pellets were thawed on ice and suspended in 600 µl 0.5 mg/ml lysozyme, in TE (10 mM Tris, 1 mM ethylenediamine tetraacetic acid), pH 8.0. Cells were lysed by the addition of 60 µl 10% SDS and heating at 64°C for 2 minutes, followed by the addition of 66 µl 1 M sodium acetate, pH 5.2 and 750 µl ROTI aqua-phenol (Roth #A980.3). After heating at 64°C with gentle inversion several times for 6 minutes and incubation on ice for 5 minutes, phases were separated by centrifugation at 13,000 rpm for 15 minutes at 4°C. The aqueous phase was re-extracted with 750 µl chloroform, followed by centrifugation at 13,000 rpm for 12 minutes at 4°C. RNA was then precipitated with 2 volumes of 30:1 mix (ethanol:3M sodium acetate, pH 6.5) overnight at -20°C, harvested by centrifugation at 13,000 rpm for 30 minutes, and washed once with cold 75% ethanol, dried, and resuspended with RNase-free water.

Detection of TIS/TTS sites and associated ORFs. For detection of TIS/TTS, we adapted previous peak detection methods[23,25]. All programming scripts are available at https://github.com/RickGelhausen/StartStopFinder (version 1.0.0). The lack of support for

TTS libraries at the time provided the impetus to develop our own tool rather than previously published scripts[26]. Our method also avoids issues with overlapping start codons that were not addressed in these scripts. First, coverage files (normalized per million reads) were generated by HRIBO[27] using different single-nucleotide mapping strategies (3′ or 5′ end) and read lengths (27-33 nt). Metagene analysis of ribosome occupancy at start or stop codons was performed as described previously[21] to determine the offset from the start or stop codon giving highest read density. For this analysis, windows of 100 nucleotides upstream and 400 nucleotides downstream of the start or 250 nucleotides upstream as well as downstream of the stop codon were extracted for each annotated coding feature. Mapped reads were separated by read length, and depending on the specific coverage display approach, either the 3' or the 5' end of each read was assigned to the extracted windows. The resulting read density was then normalized over the window size. In the displayed graphs a narrower window around start/stop codons are shown. Read length and offsets (see below) were determined independently for each experiment (*i.e*, TIS(Ret) and TIS/Onc).

These parameters were used for TIS peak detection and ORF prediction. All start codons (ATG/TTG/GTG) were collected and intervals of 5 nt, selected based on metagene analysis, that span around each start codon were generated. Intervals were then shifted by the previously determined offset. For each position in the coverage file that overlapped a given interval, the peak height was defined as the sum of the overlapping positions that had a read count higher than 5 reads. For each interval that had a non-zero peak height, the next in-frame stop codon was identified to generate a corresponding ORF (of any length). Associated TE and RPKM values were calculated. A similar method was used for detection of stop codons with TTS data, except potential sORFs with stop codons downstream of annotated genes within 25 nt were excluded to reduce signals from ribosome stop codon readthrough.

The following coverage files and offsets were used for start codon detection: TIS(Ret): 3′ end read coverage, +16 nt offset, 31 + 32 nt read length coverage files; TIS/TTS(Onc/Api): 3′ end of read coverage, +17 nt offset, 32 nt read length coverage files. For peak detection in TTS libraries using the following data: TTS-Mono: 3′ end of read coverage, +13 nt offset, 29 nt read length coverage files; TTS(Di): 3′ end of read coverage, +13 nt offset, 58 nt read length coverage files or 5′ end of read coverage, -45 nt offset, 58 nt read length coverage files. For start codons, offset positions represent the 16th nt after the first nt of NTG (*e.g.*, for + 16 nt).

For stop codons, offset positions represent the 13th nt after the first nt of the stop codon (*e.g.*, for + 13 nt). Predictions were generated with the same parameters as for TIS detection, except an interval of 5 nt around the offset nucleotide was used.

TIS and TTS sites were classified as follows: Annotated: within 3 nt (up or downstream) of an annotated Start codon; Internal in-frame: within an annotated ORF, N-terminal truncation/Internal start site and same stop codon; Internal out-of-frame: within an annotated ORF, different reading frame; Unannotated - outside of annotated features.

sORF predictions based on the third experiment (TIS(Onc) and TTS) were generated using only a single replicate available at the time. To validate the method and determine the robustness of TTS detection, we generated two additional replicates and used these for automated predictions of annotated and novel sORFs (**Supplementary Data file 12**, predictions summarized in **Supplementary Data file 13**). This data was also used to manually curate TTS for longer genes (*e.g.*, phase-variable, **Fig. 4 & Supplementary Data file 13**).

Manual curation of Ribo-seq/TIS/TTS-based sORF predictions. The 421 filtered sORF predictions were manually inspected (**Supplementary Data file 6**) based on RNA-seq/Ribo-seq coverage files in a genome browser. We used criteria previously published for prokaryotic Ribo-seq data[28–30], outlined in the **Supplementary Table 1** below. Briefly, coverage files for replicate 1 of each experiment (for mapping and read-length information see **Supplementary Table 1** below) were loaded together in IGB (Integrated Genome Browser[31], https://www.bioviz.org/) along with ORF, sRNA, and TSS annotations[1,2], a minimal RBS (AAGG) track, and dRNA-seq data[1]. Predictions were inspected by two researchers (KF, SLS) together. The final list of high-confidence 42 novel sORFs is based on their assessment of coverage patterns. Overall, our selected high-confidence sORFs were not required to meet all the below-listed criteria. For example, sORFs within operons do not necessarily have their own transcriptional start site as they are typically co-transcribed with the upstream gene. However, we required at least two independent indications for assigning a predicted sORF to our high-confidence list. Furthermore, candidates with some coverage, but which did not meet most criteria in **Supplementary Table 1**, were discarded as our aim was to provide high-confidence candidates. For additional guidance, information regarding

why the individual sORF predictions were classified as high-confidence CJsORFs or rejected are given in **Supplementary Data file 6**.

1. Translational efficiency (TE): the **Ribo-seq signal** was generally required to be **comparable to or higher** than the **transcriptome library** (*i.e.,* TE approx. 1) **across the ORF** (i.e., not arising from high density at only a single position).
2. Coverage shape: ORFs with **higher Ribo-seq coverage near the start codon** and/or **restricted within ORF boundaries** (and excluded from 5′/3′UTRs) were prioritized, even if the TE was <1. Internal in-frame or internal-out-of-frame candidates generally showed changes in coverage profile compared to the parental ORF, *e.g.,* an increase in Ribo-seq reads at the TIS region of the overlapping sORF.
3. TIS peaks: the **offset** with respect to the start codon (**+/- 2 nt** around the ideal offset of +16 or +17 nt based on metagene analysis for 3' mapping of Ret or Onc, respectively) as well as **peak height and enrichment** (vs. the Ribo-seq library) were evaluated. In addition, we assessed the enrichment of TIS peaks **with respect to surrounding noise**: the actual TIS peak needed to show not only enrichment vs. the corresponding Ribo-seq library at the same position but also to surrounding low noise in the TIS library. Only in regions that do **not show background** noise, **small TIS peaks** were considered as true.
4. TTS peaks: the **offset of +13 nt (using 3' mapping)** with respect to the stop codon **+/- 2 nt** (based on metagene analysis) as well as peak height and presence of a TIS(Onc) peak were considered. TTS peaks **with a TIS(Onc) peak** at the same position were **discarded**. We also **evaluated local enrichment** compared to the **surrounding noise** (similar to described above for TIS peaks), as well as whether the peak might reflect stop codon read through. In case of a noisy surrounding region, the TTS peak needs to be enriched or is otherwise not considered as a true TTS peak. Similarly, only for regions with no noise very small TTS peaks (around 5 reads) were considered.
5. Corresponding **start codon identification (for TTS based predictions)**: Prediction of the full ORF is not possible by the sole use of TTS data and thus requires manual inspection of TIS and Ribo-seq data, as well as inspection for a **putative RBS**. For all

sORF predictions, the presence of a potential RBS and **transcriptional start site** (TSS) position were also considered as supporting criteria in the decision whether a sORF is true.

Supplementary Table 1: Guidelines for manual curation of Ribo-seq-based datasets of *C. jejuni*. Criteria for including or not including a predicted sORF in the final high confidence list are indicated for Ribo-seq, TIS and TTS profiling. In addition, other investigated features as well as the specific read-length restrictions and mapping approaches of the different libraries/datasets are listed. N/A: not applicable; TE: translational efficiency.

Supplementary Table 1: Guidelines for manual curation

	Mapping / Read length	Feature	sORF Accepted/prioritized, if:	sORF Discarded/unfavored, if:
**Ribo-seq**	Full-read / all read lengths	**Magnitude / enrichment of coverage**	● Ribo-seq coverage is comparable to or higher than the transcriptome library (*i.e.,* TE approx. 1 or higher) over the ORF.	● Ribo-seq coverage is substantially lower than the transcriptome library (*i.e.,* TE < 0.5).<br>● high Ribo-seq coverage arises from localized high read density.
		**Shape of coverage**	● Ribo-seq coverage spans the ORF and is enriched near the start codon/5' end of sORF.<br>● Ribo-seq coverage is restricted within ORF boundaries (excluding the last/first ~15 nt of 5'/3'UTRs), while RNA-seq coverage extends into UTRs.	● Ribo-seq coverage does not overlap the predicted ORF/partially overlaps.<br>● high Ribo-seq coverage arises from localized high read density (see above).
**TIS profiling**	3' end /<br><br><u>TIS(Ret):</u><br>31+32 nt<br>Offset: +16 nt<br><u>TIS(Onc):</u><br>32 nt<br>Offset: +17 nt	**TIS library peak position**	● the TIS peak is located +/- 2 nt around the ideal offset nt (from the start codon) that was determined by metagene analysis.	● the TIS peak is located > 2 nt upstream or downstream of the ideal offset nt (from the start codon) that was determined by metagene analysis.
		**TIS library peak enrichment**	● the TIS peak is enriched vs. peaks in the Ribo-seq library at the same position.<br>● the TIS peak is more prominent than surrounding noise in the same library.	● the TIS peak is not enriched compared to the Ribo-seq library at the same position.<br>● the TIS peak is surrounded by peaks of similar or greater height.
**TTS profiling**	<u>TTS(Mono):</u><br>3' end / 29 nt	**TTS library peak position**	● the TTS peak is located +/- 2 nt around the ideal offset (from the stop codon) based on metagene analysis.	● the TTS peak is located upstream or downstream > 2 nt around the ideal offset based on metagene analysis.

**TTS profiling**	Offset: +13 nt<br><br><u>TTS(Di):</u><br>5' and 3' end / 58 nt<br>Offsets:<br>5': -45 nt<br>3': +13 nt	**Stop codon read-through**	• in addition to being at the expected position (+13 nt), the region surrounding the TTS peak does not contain additional noisy peaks, other than those present immediately downstream that can be attributed to stop-codon readthrough.	• the TTS peak is in a noisy region.<br>• the TTS peak is spaced too far from the stop codon (> 17 nt downstream).
		**Peak enrichment**	• the TTS peak shows enrichment compared to the same position in the Ribo-seq library.<br>• the TTS peak shows enrichment vs. surrounding noise in the same TTS library.<br>• low TTS peaks (~ 5 reads) in noise-free regions are considered as true peaks.	• no enrichment is detected for the TTS peak compared to the Ribo-seq library at the same genomic position.<br>• a TIS peak is detected at the same position.<br>• a TTS peak with similar height is observed in the surrounding region in the same TTS library.
		**Start of ORF**	• the predicted sORF also has a potential start codon (AUG, UUG, GUG) supported by Ribo-seq and/or TIS profiling data.	• a potential start codon cannot be identified from Ribo-seq or TIS profiling coverage.
**Additional features / considerat-ions**	N/A	**RBS motif**	• the predicted sORF has at least a minimal RBS motif (AAGG) starting ~ 10 nt upstream of the start codon.	• no RBS-like sequence can be identified.<br>• an RBS-like sequence with suboptimal spacing (starting at ≥ 14 nt or ≤ 6 nt) to start codon is present (could also mean that a different start codon is used).
	N/A	**Transcription start site**	• a transcriptional start site is detected by dRNA-seq ([1,2]) upstream of the sORF.	• N/A
	N/A	**For internal/ overlapping sORFs**	• coverage is not restricted to the sORF, but there is indication that the coverage pattern is caused by the parental/ overlapping ORF.<br>• a change of coverage pattern (*e.g.*, increase of coverage around the start codon of the internal ORF) was observed.<br>• TIS peak indicates internal translation initiation.	• no increase in coverage/change in pattern is observed compared to the parental ORF.<br>• coverage seems to originate only from the ~ 15 nt that extends into the 3'UTR of the parental ORF.<br>• coverage is only present for the internal in-frame ORF and not the annotated/ parental ORF. This might indicate that the parental ORF instead requires re-annotation.

Mass spectrometry based proteomics. For preparation of soluble *C. jejuni* protein extracts, cells were disrupted in a FastPrep Homogenizer (MP-Biomedicals) for 30 s at 6.5 m/s^2 followed by incubation on ice for 4 min. This procedure was repeated two times. Immediately after the third disruption step, the lysate was centrifuged two times for 15 min (12,000 g at 4°C) to remove all cell debris and insoluble and aggregated proteins. Protein concentration was determined as described previously[32] using the Roti-Nanoquant assay (Roth, Karlsruhe, Germany). Two different techniques for pre-fractionation of proteins were applied: (i) separation of soluble proteins by one dimensional (1D) SDS-PAGE and in-gel digestion with trypsin or chymotrypsin (see "gel-based approach" described in (Fuchs et al. 2021), or (ii) fractionation of proteins on a GELFREE 8100 fractionator (Expedeon) and trypsin digestion. For the gel-based approach, we separated 36 µg protein crude extracts on 15% polyacrylamide gels. For in-gel digestion with chymotrypsin, an enzyme/protein ratio of 1:10 was applied for 16 h at pH 8 and 30°C.

For protein fractionation on a GELFREE 8100 fractionator, 200 µg soluble proteins were separated according to manufacturer's instructions (Expedeon) using 10% Tris acetate cartridges. In addition to the samples recommended by the manufacturer (Expedeon), a sample 220 min after starting fractionation was taken. Protein digestion was performed in protein low binding tubes (Eppendorf, Hamburg, Germany) using the Single-Pot Solid-Phase-enhanced Sample Preparation technique used previously[33] with some modifications as follows. Magnetic beads were washed three times with MilliQ water before use. 30 µg beads were added to each sample, which was adjusted to pH <5 using 5% formic acid. To bind the proteins to the beads, a fourfold volume of acetonitrile was added and samples were mixed horizontally at room temperature. After two to three hours, additional 30 µg beads were given to each sample which were incubated as aforementioned overnight. Samples were centrifuged (13,000 g, 5 min, room temperature), placed on a magnetic rack and the supernatant was removed. Proteins bound to magnetic beads were washed two times with ethanol and incubated with 50 mM DTT in 50 mM NH_4HCO_3, 1 mM $CaCl_2$ for 30 min at 60°C. Subsequently, iodoacetamide was added to each sample with a final concentration of 120 mM and incubated for 20 min at room temperature. For protein digestion, trypsin was applied with an enzyme/protein ratio of 1:50. After incubation for 12 h (800 rpm, 37 °C), samples were adjusted to pH <5 using 5% formic acid, centrifuged and peptides bound to

magnetic beads were washed two times with acetonitrile. Peptide elution was performed in two steps. First, magnetic beads were treated with 20 µl 2% DMSO for 30 min, centrifuged and the supernatant was transferred to a new tube. In a second step, the beads were incubated with 20 µl 0.065% formic acid, 500 mM KCl in 30% acetonitrile for 30 min and centrifuged. The supernatants of both elution steps were combined, vacuum dried and stored at -20°C. Peptide desalting was performed as done previously[32]. ZipTips (C18, Merck Millipore, Billerica, MA, USA) were conditioned with 50% acetonitrile twice and equilibrated three times with 0.1% FA in 5% acetonitrile. Next, 10 µL of each peptide fraction (dissolved in 20 µL 0.1% FA in 5% acetonitrile for 60 min) were loaded on the C18 matrix of the tip 10 times by aspiration. Peptides were eluted three times by aspirating five times with 0.1% FA in 60% acetonitrile in a new microfuge tube. Samples were then dried in a speedvac.

Peptide fractions were analyzed using the Orbitrap Fusion MS coupled to a Dionex Ultimate 3000 nHPLC system (Thermo Fisher Scientific Inc., Waltham, Massachusetts, USA) as described by Fuchs *et al.*[32] with some modifications as follows. Primary Scans were performed in the profile modus scanning an m/z of 350 - 1,700 with a resolution (full width at half maximum at m/z 400) of 120,000 and a lock mass 445.12003. Using Xcalibur software (Thermo Fisher Scientific Inc., San Jose, SA, USA), the mass spectrometer was controlled and operated in the "top 20" mode, selecting the 20 most abundant MS ions for fragmentation. Primary ions (±10 ppm) were selected by the quadrupole (isolation window: 1.6 m/z), fragmented by CID (collision energy 35%, activation Q 0.25) and analyzed in the ion trap with an exclusion time of 20 s. The charges of MS ions used for fragmentation were 2 to 6 for trypsin or 1 to 6 for chymotrypsin.

For identification of small proteins in *C. jejuni* based on MS/MS data, we used the bacterial proteogenomics workflow described previously (SALT & Pepper; https://gitlab.com/s.fuchs/pepper;[32]). This fully automated workflow includes protein database generation, database searching, peptide-to-genome mapping, and result interpretation. MS- and MS/MS-data of all samples were searched by MaxQuant (Max Planck Institute of Biochemistry, Martinsried, Germany, www.maxquant.org, version 1.5.2.8) against a database with *C. jejuni* annotated protein sequences from NCBI (downloaded at 01-09-2020) and sORFs that were predicted using our Ribo-seq data and a translational database (TRDB) of the full coding potential of the *C. jejuni* genome generated by six-frame

translation from stop codon to stop codon with a minimum length of 9 aa generated by SALT (https://gitlab.com/s.fuchs/pepper). For chymotrypsin, the number of missed cleavages was set to 4 and maximum charge to 7.

Co-immunoprecipitation (coIP) for investigation of protein-protein interactions. Strains carrying chromosomally epitope-tagged versions of CioA and/or CioY were used (CioA-SPA & CioY-sfGFP (stock CSS-8004) and reciprocal version CioY-SPA & CioA-sfGFP (stock CSS-6727)). Pulldown of the SPA-tagged protein was performed with an anti-FLAG antibody (Sigma-Aldrich, #F1804-1MG) bound to Protein A-Sepharose beads (Sigma-Aldrich, #P6649), and co-purification of the second sfGFP tagged protein was investigated by western blot with an anti-GFP antibody (Roche #11814460001). As a control for unspecific binding, lysates of an untagged wild-type strain (CSS-5295) as well as the corresponding sfGFP-only tagged strain (CioA-sfGFP (CSS-6725) or CioY-sfGFP (CSS-8002)) alone were also used. Lysates for coIP were prepared from ~60 OD_{600} of cells harvested at exponential phase (OD_{600} 0.6, 5000 rpm, 20 min, 4°C) which were first washed once in buffer A (20 mM TrisHCl pH 8.0, 1 mM $MgCl_2$, 150 mM KCl, 1 mM DTT) (8000 *g*, 2 min, 4°C). In parallel, 1 OD_{600} of cells was harvested for whole cell lysate analysis and boiled for 8 min at 95°C in 1× protein loading buffer. After the supernatant was discarded, cell pellets were snap frozen in liquid nitrogen and stored at -80°C until further use. Thawed cell pellets were lysed in 1 ml lysis buffer (buffer A incl. 1 mM PMSF (phenylmethylsulfonyl fluoride, Roche), 20 U DNase I (Thermo Fisher Scientific), 200 U RNase Inhibitor (moloX, Berlin) and 1% DDM (n-dodecyl-B-D-maltoside) with a FastPrep system (MP Biomedical, matrix B, 1× 4 m/s, 10 sec). Lysates were cleared by centrifugation (13,000 rpm, 10 min, 4°C) and an aliquot (1 OD_{600}) was set aside as the input/lysate control for western blot analysis. The lysate was then incubated with rotation for 30 min at 4°C with 35 µl anti-FLAG antibody. The Protein A-Sepharose beads (75 µl/sample) were washed three times with buffer A and then lysates, pre-incubated with anti-FLAG antibody, were added to the pre-washed beads and incubated for another 30 min at 4°C with rotation. The supernatant (unbound fraction) was removed after centrifugation (15,000 *g*, 1 min, 4°C). Beads with the bound proteins were washed five times with buffer A. Finally, the bound proteins were eluted by adding 400 µL 1× protein loading buffer and boiling for 8 min at 95°C with shaking at 1000 rpm. The eluate was

precipitated with a minimum 6 vol acetone overnight at -20°C. Precipitated proteins were collected by centrifugation (15,000 rpm, 1 h, 4°C), dried, and resuspended in 1× protein loading buffer. For verification of successful coIP and to investigate potential interactions, whole cell lysate (0.1 OD_{600}), lysate/input samples (0.1 OD_{600}) and the eluates (5 OD_{600}) were used for western blot analysis. Blots were probed for anti-GFP for investigation of interaction, anti-FLAG for verification of pull-down, and finally anti-GroEL as a loading control. Two independent biological replicates of each coIP experiment were performed.

Online References

1. Dugar, G. *et al.* High-resolution transcriptome maps reveal strain-specific regulatory features of multiple *Campylobacter jejuni* isolates. *PLoS Genet.* **9**, e1003495 (2013).
2. Gundogdu, O. *et al.* Re-annotation and re-analysis of the *Campylobacter jejuni* NCTC11168 genome sequence. *BMC Genomics* **8**, 162 (2007).
3. Porcelli, I., Reuter, M., Pearson, B. M., Wilhelm, T. & van Vliet, A. H. M. Parallel evolution of genome structure and transcriptional landscape in the Epsilonproteobacteria. *BMC Genomics* **14**, 616 (2013).
4. Meydan, S., Vázquez-Laslop, N. & Mankin, A. S. Genes within Genes in Bacterial Genomes. *Microbiol. Spectr.* **6**, (2018).
5. Corpet, F. Multiple sequence alignment with hierarchical clustering. *Nucleic Acids Res.* **16**, 10881–10890 (1988).
6. Clauwaert, J., Menschaert, G. & Waegeman, W. DeepRibo: a neural network for precise gene annotation of prokaryotes by combining ribosome profiling signal and binding site patterns. *Nucleic Acids Res.* **47**, e36 (2019).
7. Bailey, T. L. *et al.* MEME SUITE: tools for motif discovery and searching. *Nucleic Acids Res.* **37**, W202-8 (2009).
8. Yu, N. Y. *et al.* PSORTb 3.0: improved protein subcellular localization prediction with refined localization subcategories and predictive capabilities for all prokaryotes. *Bioinformatics* **26**, 1608–1615 (2010).
9. Katoh, K. & Standley, D. M. MAFFT multiple sequence alignment software version 7: improvements in performance and usability. *Mol. Biol. Evol.* **30**, 772–780 (2013).
10. Price, M. N., Dehal, P. S. & Arkin, A. P. FastTree: computing large minimum evolution trees with profiles instead of a distance matrix. *Mol. Biol. Evol.* **26**, 1641–1650 (2009).
11. Parkhill, J. *et al.* The genome sequence of the food-borne pathogen *Campylobacter jejuni* reveals hypervariable sequences. *Nature* **403**, 665–668 (2000).
12. Carver, T., Thomson, N., Bleasby, A., Berriman, M. & Parkhill, J. DNAPlotter: circular and linear interactive genome visualization. *Bioinformatics* **25**, 119–120 (2009).
13. Buels, R. *et al.* JBrowse: a dynamic web platform for genome visualization and analysis. *Genome Biol.* **17**, 66 (2016).
14. Bury-Moné, S. *et al.* Presence of active aliphatic amidases in *Helicobacter* species able to colonize the stomach. *Infect. Immun.* **71**, 5613–5622 (2003).
15. Dugar, G. *et al.* The CsrA-FliW network controls polar localization of the dual-function flagellin mRNA in *Campylobacter jejuni*. *Nat. Commun.* **7**, 11667 (2016).
16. Cameron, A. & Gaynor, E. C. Hygromycin B and apramycin antibiotic resistance cassettes for use in *Campylobacter jejuni*. *PLoS ONE* **9**, e95084 (2014).
17. Ribardo, D. A., Bingham-Ramos, L. K. & Hendrixson, D. R. Functional analysis of the RdxA and RdxB nitroreductases of *Campylobacter jejuni* reveals that mutations in *rdxA* confer metronidazole resistance. *J. Bacteriol.* **192**, 1890–1901 (2010).
18. Dugar, G. *et al.* CRISPR RNA-Dependent Binding and Cleavage of Endogenous RNAs by the *Campylobacter jejuni* Cas9. *Mol. Cell* **69**, 893-905.e7 (2018).
19. Alzheimer, M. *et al.* A three-dimensional intestinal tissue model reveals factors and small regulatory RNAs important for colonization with *Campylobacter jejuni*. *PLoS Pathog.* **16**, e1008304 (2020).
20. Oh, E. *et al.* Selective ribosome profiling reveals the cotranslational chaperone action of trigger factor *in vivo*. *Cell* **147**, 1295–1308 (2011).
21. Becker, A. H., Oh, E., Weissman, J. S., Kramer, G. & Bukau, B. Selective ribosome profiling as a tool for studying the interaction of chaperones and targeting factors with nascent polypeptide chains and ribosomes. *Nat. Protoc.* **8**, 2212–2239 (2013).

22. Wiegand, I., Hilpert, K. & Hancock, R. E. W. Agar and broth dilution methods to determine the minimal inhibitory concentration (MIC) of antimicrobial substances. *Nat. Protoc.* **3**, 163–175 (2008).
23. Weaver, J., Mohammad, F., Buskirk, A. R. & Storz, G. Identifying Small Proteins by Ribosome Profiling with Stalled Initiation Complexes. *MBio* **10**, (2019).
24. Meydan, S. *et al.* Retapamulin-Assisted Ribosome Profiling Reveals the Alternative Bacterial Proteome. *Mol. Cell* **74**, 481-493.e6 (2019).
25. Gelsinger, D. R. *et al.* Ribosome profiling in archaea reveals leaderless translation, novel translational initiation sites, and ribosome pausing at single codon resolution. *Nucleic Acids Res.* **48**, 5201–5216 (2020).
26. Bartholomäus, A. *et al.* smORFer: a modular algorithm to detect small ORFs in prokaryotes. *Nucleic Acids Res.* **49**, e89 (2021).
27. Gelhausen, R. *et al.* HRIBO: high-throughput analysis of bacterial ribosome profiling data. *Bioinformatics* **37**, 2061–2063 (2021).
28. Gelhausen, R. *et al.* RiboReport - benchmarking tools for ribosome profiling-based identification of open reading frames in bacteria. *Brief. Bioinformatics* **23**, (2022).
29. Hadjeras, L. *et al.* Revealing the small proteome of *Haloferax volcanii* by combining ribosome profiling and small-protein optimized mass spectrometry. *microLife* **4**, uqad001 (2023).
30. Hadjeras, L. *et al.* Unraveling the small proteome of the plant symbiont *Sinorhizobium meliloti* by ribosome profiling and proteogenomics. *microLife* **4**, uqad012 (2023).
31. Freese, N. H., Norris, D. C. & Loraine, A. E. Integrated genome browser: visual analytics platform for genomics. *Bioinformatics* **32**, 2089–2095 (2016).
32. Fuchs, S. *et al.* Towards the characterization of the hidden world of small proteins in *Staphylococcus aureus*, a proteogenomics approach. *PLoS Genet.* **17**, e1009585 (2021).
33. Hughes, C. S. *et al.* Ultrasensitive proteome analysis using paramagnetic bead technology. *Mol. Syst. Biol.* **10**, 757 (2014).
