# Supplementary material for: Complementary Ribo-seq approaches map the translatome and provide a small protein census in the foodborne pathogen Campylobacter jejuni
Source: Nat Commun. 2025 Mar 30;16:3078. doi: 10.1038/s41467-025-58329-w (PMC11955535; doi:10.1038/s41467-025-58329-w)
Supplement: Supplementary file 3 — Description of Additional Supplementary Files [file 41467_2025_58329_MOESM3_ESM.pdf]

File: Supplementary Data file 1
Description: Features and detection information for annotated and novel sORFs in *C. jejuni* NCTC11168.

File: Supplementary Data file 2
Description: Mass spectrometry (MS) based detection of annotated and novel *C. jejuni* ORFs.

File: Supplementary Data file 3
Description: Potential leaderless *C. jejuni* ORFs.

File: Supplementary Data file 4
Description: Re-annotation of *C. jejuni* start codons based on Ribo-seq and TIS profiling data.

File: Supplementary Data file 5
Description: All changes suggested for the NCTC11168 annotation based on translatomics approaches.

File: Supplementary Data file 6
Description: Overview of manual curation of automated and filtered sORF predictions.

File: Supplementary Data file 7
Description: Putative start-stop sites identified by TIS(Ret) and TIS(Onc).

File: Supplementary Data file 8
Description: Epsilonproteobacteria genome sequences used for conservation analysis.

File: Supplementary Data file 9
Description: Bacterial strains used in this study.

File: Supplementary Data file 10
Description: Plasmids used in this study.

File: Supplementary Data file 11
Description: Oligonucleotides.

File: Supplementary Data file 12
Description: Sequencing dataset statistics.

File: Supplementary Data file 13
Description: Overview of three replicates for TIS(Onc) and TTS(Mono/Di) predictions of novel CJsORFs and TTS-candidates in *C. jejuni* NCTC11168.
