# Supplementary material for: Complementary Ribo-seq approaches map the translatome and provide a small protein census in the foodborne pathogen Campylobacter jejuni
Source: Nat Commun. 2025 Mar 30;16:3078. doi: 10.1038/s41467-025-58329-w (PMC11955535; doi:10.1038/s41467-025-58329-w)
Supplement: Supplementary file 17 — Reporting Summary [file 41467_2025_58329_MOESM17_ESM.pdf]

nature portfolio

Corresponding author(s): Cynthia M. Sharma

Last updated by author(s): Mar 5, 2025

Reporting Summary

Nature Portfolio wishes to improve the reproducibility of the work that we publish. This form provides structure for consistency and transparency in reporting. For further information on Nature Portfolio policies, see our Editorial Policies and the Editorial Policy Checklist.

Statistics

For all statistical analyses, confirm that the following items are present in the figure legend, table legend, main text, or Methods section.

n/a	Confirmed	
☐	☒	The exact sample size (*n*) for each experimental group/condition, given as a discrete number and unit of measurement
☐	☒	A statement on whether measurements were taken from distinct samples or whether the same sample was measured repeatedly
☐	☒	The statistical test(s) used AND whether they are one- or two-sided<br>*Only common tests should be described solely by name; describe more complex techniques in the Methods section.*
☒	☐	A description of all covariates tested
☒	☐	A description of any assumptions or corrections, such as tests of normality and adjustment for multiple comparisons
☐	☒	A full description of the statistical parameters including central tendency (e.g. means) or other basic estimates (e.g. regression coefficient) AND variation (e.g. standard deviation) or associated estimates of uncertainty (e.g. confidence intervals)
☐	☒	For null hypothesis testing, the test statistic (e.g. *F*, *t*, *r*) with confidence intervals, effect sizes, degrees of freedom and *P* value noted<br>*Give P values as exact values whenever suitable.*
☒	☐	For Bayesian analysis, information on the choice of priors and Markov chain Monte Carlo settings
☒	☐	For hierarchical and complex designs, identification of the appropriate level for tests and full reporting of outcomes
☒	☐	Estimates of effect sizes (e.g. Cohen's *d*, Pearson's *r*), indicating how they were calculated

Our web collection on statistics for biologists contains articles on many of the points above.

Software and code

Policy information about availability of computer code

Data collection	Western blot images were acquired with an ImageQuantTM LAS 4000 (version 1.3, build 1.3.0.134).<br><br>The reference genome sequence NC_002163.1 (ASM908v1) and annotation was recovered from NCBI (2014-03-20). Published differential RNA-seq data (Dugar et al. 2013, PMID: 23696746) was retrieved from GEO (Accession GSE38883; https://www.ncbi.nlm.nih.gov/geo/query/acc.cgi?acc=GSE38883). E. coli CydA and CydX structures were recovered from PDB (6RKO; https://doi.org/10.2210/pdb6rko/pdb). Cells were imaged with a laser scanning Leica TCS SP5 II confocal microscope (Leica Microsystems) with LAS AF Software (version 2.7.3.9723). Genomes for conservation analysis were downloaded from the NCBI GenBank or RefSeq databases (see Supplementary Data file 8 for accessions, accessed before Jan 2023) (RRID:SCR_003496) (RRID:SCR_002760).<br><br>RNA-seq libraries were sequenced on a NextSeq500. Publicly available assembled genome sequences for 17 species were downloaded from the National Center for Biotechnology Information GenBank or RefSeq databases (before Jan 2023). MLST sequence types and clonal complexes were obtained by scanning the genome sequences against the PubMLST database using mlst (v2.23.0) (https://github.com/tseemann/mlst).
Data analysis	Western blot images and confocal micrographs were prepared with ImageJ (version 1.53f). Densitometry analyses were conducted using AIDA image analysis software (v5.0, build 1182, Raytest, Germany). Statistical analyses were performed with Graphpad Prism v 7.04. Venn diagrams were generated using Venny (version 2.1.0, https://bioinfogp.cnb.csic.es/tools/venny/).<br><br>Ribo-seq data was analysed using HRIBO (version 1.4.4, PMID: 33175953, https://github.com/RickGelhausen/HRIBO) and StartStopFinder

(version 1.0.0, https://github.com/RickGelhausen/StartStopFinder). Software within HRIBO include: snakemake (RRID:SCR_003475, PMID: 29788404), bioconda (RRID:SCR_018316, PMID: 29967506), docker, cutadapt (version 2.1, RRID:SCR_011841), segemehl (version 0.3.4, RRID:SCR_005494, PMID: 19750212), SAMtools (version 1.9, RRID:SCR_002105, PMID: 19505943). The adapted version of REPARATION (version 1.0.9) is available at https://github.com/RickGelhausen/REPARATION_blast. RNA-seq coverage was visualized with IGB (version 9.1.10, https://www.bioviz.org/, PMID: 27153568). CampyBrowse is based on JBrowse (version 2.11.1), PMID: 27072794, RRID:SCR_001004). Sequences were aligned using multalin (PMID: 2849754, http://multalin.toulouse.inra.fr/multalin/).

Nucleotide motifs were predicted using MEME (version 5.4.1, RRID:SCR_001783, PMID: 19458158, https://meme-suite.org/meme/). Amino acid sequences were analyzed for localization signals using PSORTb (version 3.0, PMID: 20472543, RRID:SCR_007038, https://www.psort.org/psortb/). Genome-based small protein predictions were performed with ranSEPs, http://ranseps.crg.es/, PMID: 30796087) and smORFfinder (PMID: 33290720). Genome quality was estimated using checkM (v1.1.3) (PMID: 25977477) (RRID:SCR_016646). MLST and phylogenetic analysis were performed with mlst (v2.23.0) (PMID: 30345391) (RRID:SCR_010245). Maximum-likelihood phylogenetic trees were constructed using FastTree (v2.1.10) (PMID: 19377059). Assemblies were aligned against reference genomes with MUMmer (v3.23) (PMID: 14759262). SNP calling was performed using SNP-sites (v2.5.1) (PMID: 28348851) (RRID:SCR_022265). Repetitive genome regions were identified using TRF (v4.07b) (PMID: 9862982) (RRID:SCR_022193). Blastn and tblastn (v2.11.0+) (PMID: 20003500) (RRID:SCR_001598) (RRID:SCR_001598) (RRID:SCR_001010) were used for sORF detection. sORFs were aligned using MAFFT version 7 (v7.505) (PMID: 23329690) (RRID:SCR_011811). Annotated sORF homologs were detected by BLASTp at NCBI (RRID:SCR_001010). Genome rings were prepared using the DNA plotter tool within the Artemis package (version v18.2.0, PMID: 18990721).

For MS data analysis, the following tools were used: SALT & Pepper (https://gitlab.com/s.fuchs/pepper; PMID: 34061833) and MaxQuant (Max Planck Institute of Biochemistry, Martinsried, Germany, www.maxquant.org, version 1.5.2.8, RRID:SCR_014485). Protein structures were visualized in Pymol (version 2.5.2, RRID:SCR_000305). Protein structure predictions were performed with Alphafold2-multimer at Colabfold (https://colab.research.google.com/github/sokrypton/ColabFold/blob/main/AlphaFold2.ipynb#scrollTo=KK7X9T44pWb7, accessed 2022-03-26, PMID: 35637307).

For manuscripts utilizing custom algorithms or software that are central to the research but not yet described in published literature, software must be made available to editors and reviewers. We strongly encourage code deposition in a community repository (e.g. GitHub). See the Nature Portfolio guidelines for submitting code & software for further information.

Data

Policy information about availability of data

All manuscripts must include a data availability statement. This statement should provide the following information, where applicable:

- Accession codes, unique identifiers, or web links for publicly available datasets
- A description of any restrictions on data availability
- For clinical datasets or third party data, please ensure that the statement adheres to our policy

Ribo-seq data have been deposited at the NCBI Gene expression Omnibus (GEO) under the Accession GSE208756 (https://www.ncbi.nlm.nih.gov/geo/query/acc.cgi?acc=GSE208756). The mass spectrometry proteomics data have been deposited to the ProteomeXchange Consortium (http://proteomecentral.proteomexchange.org) via the PRIDE partner repository (https://www.ebi.ac.uk/pride/archive) with the dataset identifier PXD036790 (https://proteomecentral.proteomexchange.org/cgi/GetDataset?ID=PXD036790). Differential RNA-seq data (Dugar et al., 2013) was recovered from GEO (GSE38883; https://www.ncbi.nlm.nih.gov/geo/query/acc.cgi?acc=GSE38883). The sRNA and 5'UTR annotation was generated from data from Dugar et al. 2013 (PMID: 23696746) and is available for download at GEO (GSE208756; https://www.ncbi.nlm.nih.gov/geo/query/acc.cgi?acc=GSE208756). E. coli CydA and CydX structures were recovered from PDB (6RKO; https://doi.org/10.2210/pdb6rko/pdb). The CampyBrowse resource is available at http://www.bioinf.uni-freiburg.de/~ribobase/campybrowse/overview.html. All other data supporting the findings of this study are available within the article and its supplementary and source data files.

Research involving human participants, their data, or biological material

Policy information about studies with human participants or human data. See also policy information about sex, gender (identity/presentation), and sexual orientation and race, ethnicity and racism.

Reporting on sex and gender	N/A
Reporting on race, ethnicity, or other socially relevant groupings	N/A
Population characteristics	N/A
Recruitment	N/A
Ethics oversight	N/A

Note that full information on the approval of the study protocol must also be provided in the manuscript.

Field-specific reporting

Please select the one below that is the best fit for your research. If you are not sure, read the appropriate sections before making your selection.

☒ Life sciences ☐ Behavioural & social sciences ☐ Ecological, evolutionary & environmental sciences

For a reference copy of the document with all sections, see nature.com/documents/nr-reporting-summary-flat.pdf

Life sciences study design

All studies must disclose on these points even when the disclosure is negative.

Sample size	All western blots and motility assays shown are representative of at least 2 independent biological replicates. Ribosome profiling datasets were generated for three independent biological samples (cultures). Sample sizes were selected based on prior experience with similar experimental setups to determine statistical significance as previously published in studies from our lab.
Data exclusions	No data was excluded.
Replication	Ribo-seq/novel sORF prediction: for Ribo-seq, TIS profiling and TTS profiling datasets, three independent biological replicates were performed. Novel CJsORFs were required to be predicted in at least two replicates with the exception of the TTS profiling where only one replicate was available at the time. However, the two additional replicates were investigated for all CJsORFs and genes for which a stop codon re-annotation was suggested, which is summarized in Supplementary Data file 13.<br><br>WB analysis: all examined protein samples were analyzed in two replicates (successful reproducibility).<br><br>Motility assay was performed in three biological replicates and each replicate included three technical replicates (successful reproducibility).<br><br>coIP experiments: Each way of the reciprocal coIP versions was performed in two biological replicates (successful reproducibility).<br><br>Screenshots of Ribo-seq data: Examples shown in the manuscript are representative of three independent replicates (successful reproducibility).
Randomization	Randomization was not relevant for the study as corresponding samples were prepared together.
Blinding	Blinding was not relevant as none of the readouts was subjective.

Reporting for specific materials, systems and methods

We require information from authors about some types of materials, experimental systems and methods used in many studies. Here, indicate whether each material, system or method listed is relevant to your study. If you are not sure if a list item applies to your research, read the appropriate section before selecting a response.

Materials & experimental systems

n/a	Involved in the study	
☐	☒	Antibodies
☒	☐	Eukaryotic cell lines
☒	☐	Palaeontology and archaeology
☒	☐	Animals and other organisms
☒	☐	Clinical data
☒	☐	Dual use research of concern
☒	☐	Plants

Methods

n/a	Involved in the study	
☒	☐	ChIP-seq
☒	☐	Flow cytometry
☒	☐	MRI-based neuroimaging

Antibodies

Antibodies used	1) primary antibody: monoclonal mouse anti-FLAG; 1:1,000; Sigma-Aldrich; #F1804-1MG; RRID:AB_262044<br>2) primary antibody: monoclonal mouse anti-GFP; 1:1,000; Roche #11814460001; RRID:AB_390913<br>3) secondary antibody: polyclonal sheep anti-mouse IgG horseradish peroxidase (HRP) conjugate; 1:10,000; GE Healthcare; #RPN4201<br>4) primary antibody: antibody specific for GroEL (rabbit polyclonal); 1:10,000; Sigma-Aldrich; #G6532-5ML; RRID:AB_259939<br>5) secondary antibody: polyclonal goat anti-rabbit IgG; 1:10,000; GE Healthcare; #RPN4301; RRID:AB_2650489
Validation	All primary antibodies have been validated by the manufacturers and have been previously used for the same applications by our lab (GroEL, FLAG, GFP; e.g., Pernitzsch et al. 2021, PMID: 34290242). On each western blot an untagged wild type control was included to distinguish specific from unspecific detection.

Plants

Seed stocks	N/A
Novel plant genotypes	N/A
Authentication	N/A
